# Dietary intakes of anthocyanins and cardiometabolic health: a systematic review and meta-analysis of randomized trials and prospective cohort studies in healthy participants

**DOI:** 10.1016/j.ajcnut.2026.101304

**Published:** 2026-06-18

**Authors:** Esther E Avendano, Hannah Ellingson, Elise Digga, Andrea M Velasco Nieves, Linfei Chen, Mengling Wu, Sowmyashree H Setty, Samantha Morley, Peter Curtis, Nanguneri Nirmala, Aedín Cassidy

**Affiliations:** 1 Tufts Center for Clinical Evidence Synthesis, Institute for Clinical Research and Health Policy Studies, Tufts Medical Center, Boston, MA, USA; 2 Hirsh Health Sciences Library, Tufts University School of Medicine, Boston, MA, USA; 3 Norwich Medical School, University of East Anglia, Norwich, United Kingdom; 4 Co-Centre for Sustainable Food Systems and Institute for Global Food Security, Queen’s University, Belfast, United Kingdom

**Keywords:** anthocyanins, flavonoids, cardiovascular disease, diabetes, flow-mediated dilatation, pulse wave velocity, blood pressure, healthy individuals

## Abstract

**Background:**

Meta-analyses support anthocyanins’ cardiometabolic benefits in heterogeneous populations (including diseased), but efficacy in healthy participants remains unknown.

**Objectives:**

Across prospective cohorts, and randomized controlled trials (RCTs; ≥50 mg/d anthocyanins), we assessed impacts on surrogate cardiometabolic biomarkers and clinical endpoints.

**Methods:**

Database searches (PubMed, Cochrane Central, Commonwealth Agricultural Bureau, and Web of Science) identified publications (1946–September 2024) that reported anthocyanin intake (or data, from which anthocyanin intake could be calculated), and cardiovascular disease (CVD) risk or cardiometabolic biomarkers. Random-effects meta-analysis and the Grades of Recommendation, Assessment, Development, and Evaluation (GRADE) assessment followed.

**Results:**

Across 18 cohorts in 27 publications, highest compared with lowest habitual anthocyanin intake showed a risk reduction of 26% CVD incidence (4 studies), 18% myocardial infarction (MI, 5 studies), 11% type 2 diabetes mellitus (T2DM, 8 studies), 9% CVD mortality (10 studies), and 8% hypertension risk (6 studies). GRADE identified moderate evidence for CVD/MI. Stroke risk was unaffected. Across 65 RCTs, chronic anthocyanins (≥1 d) improved flow-mediated dilation (FMD) (net change: 1.41%; 95% confidence interval: 0.90%, 1.91%), acute anthocyanins (≤1 d) improved FMD (1.50%; 1.16%, 1.84%), and insulin concentrations (−2.24 pmol/L; −4.22, −0.27). GRADE identified strong evidence for acute FMD and moderate for chronic FMD. Lipids, blood pressure, glucose, and homeostatic model assessment for insulin resistance were unaffected. In the subanalysis, by anthocyanin type, cyanidin improved chronic FMD (1.62%; 1.21%, 2.04%), whereas delphinidin improved acute FMD (1.69%; 1.20%, 2.18%), chronic FMD (1.59%; 1.21%, 2.04%), chronic pulse wave velocity (–0.33; –0.62, –0.05), insulin (–2.32 pmol/L; –4.39, –0.26), and triglycerides (–0.10 mmol/L; –0.17, –0.03).

**Conclusions:**

Higher anthocyanin intake lowers CVD, MI, hypertension, and T2DM risk in cohorts, whereas RCTs show that dietarily achievable anthocyanin intakes (as low as 50 mg/d) improve atherosclerosis and metabolic syndrome biomarkers in healthy participants. These findings provide comprehensive unifying evidence that supports the inclusion of anthocyanins within evidence-based nutrition guidelines for CVD prevention.

This review was registered at PROSPERO database as CRD420251015416.

## Introduction

There has been long-term interest in the cardioprotective effects of dietary flavonoids and their subclasses [[1], [2], [3], [4], [5], [6], [7]]. Among these, anthocyanins, glycosylated anthocyanidin pigments abundant in red/blue fruits, have shown consistent inverse associations with coronary artery disease (CAD) [[8], [9], [10]], cardiovascular disease (CVD) [9,10], type 2 diabetes mellitus (T2DM) [11], and, in an umbrella review, hypertension and T2DM [12]. These findings have prompted randomized controlled trials (RCTs) to extensively characterize mechanisms through which anthocyanin intake may influence cardiometabolic risk.

Across RCTs and meta-analyses, anthocyanins from diverse foods and purified extracts have improved anthropometric measures [13,14], glycemic outcomes [13,15,16], inflammatory mediators [10,[17], [18], [19], [20]], and total, LDL cholesterol, and HDL cholesterol, as well as triglycerides [10,15,21,22]. Improvements in endothelial function [23,24], arterial stiffness [24], and blood pressure (BP) [25] have also been reported. Although direct longitudinal evidence linking these short-term improvements with incident CAD, CVD, or T2DM is lacking, these pathways are central to disease development and provide a plausible mechanistic basis for the favorable population-based data.

However, interpretation of existing RCT meta-analyses is constrained by several factors. First, although observational data suggest that habitual intakes of 50 mg/d are associated with meaningful CAD risk reduction [5% relative risk (RR) reduction across 4 large cohorts [8]], many earlier meta-analyses included RCTs delivering substantially lower doses—commonly <50 mg/d [14,21], often <10 mg/d [10,13,19,23,24], or lacking accurate anthocyanin content data [10,23]. Given that meta-analyses indicate biomarker effects at much higher doses (e.g., ≥100 mg/d for waist circumference[26], ≥160 mg/d for triglycerides[27], ≥300 mg/d to ≥320 mg/d for inflammatory mediators such as high-sensitivity C-reactive protein (hsCRP), IL-6, TNF-α, intercellular adhesion molecule-1, and vascular cell adhesion molecule-1 [17,19], we applied a ≥50 mg/d threshold to better align RCT-derived mechanistic insights with cohort-based intake ranges. Second, previous meta-analyses routinely pooled populations with cardiometabolic diseases with healthy populations; as larger improvements typically occur in those with established disease [10,13,17], extrapolation to preventive contexts is limited. Finally, several meta-analyses restricted inclusion to purified extracts or supplements [14,16,17,21], or berry-only interventions [22], limiting assessment of anthocyanin intake irrespective of source/matrix.

To address these limitations, our meta-analyses *1*) applied a CAD-relevant intake threshold of 50 mg/d, *2*) calculated anthocyanin content where not declared, using standard databases, *3*) included all food-based and extract/supplement interventions except wine, and *4*) restricted analyses to nondiseased, healthy populations. PRISMA methodology was followed, including risk of bias assessment, evidence grading, and full meta-analysis [10,23].

By integrating findings from RCTs and prospective cohort studies, unlike prior meta-analyses [16,18,19,21,28,29], a more comprehensive assessment of anthocyanin intake, cardiometabolic biomarkers, and clinical risk reduction is provided. This approach aligns with our flavan-3-ols analysis [30], which informed US Academy of Nutrition and Dietetics guidelines [31]. We also evaluated dose-response relationships, minimum intake duration, food source and form, and subclass-specific responses, supporting more evidence-based dietary guidance and public health recommendations.

## Methods

This systematic review and meta-analysis synthesized and evaluated the association between anthocyanin intake and cardiometabolic health outcomes and risk biomarkers including CVD [e.g., CAD incidence, stroke incidence (total, ischemic, and hemorrhagic separately), and CVD mortality], hypertension, incident T2DM and a wide range of cardiometabolic health biomarkers, including flow-mediated dilation (FMD), pulse wave velocity (PWV), BP, lipids, insulin, glycated hemoglobin (HbA1c), and HOMA-IR (Supplemental Table 1). A technical expert panel was convened that served as key informants to identify issues relevant to anthocyanin, refine key questions, identify interventions of interest, and define eligibility criteria. A standard protocol was developed and registered in the PROSPERO database (https://www.crd.york.ac.uk/PROSPERO) as CRD420251015416. Our review was conducted using standard methodology and followed the PRISMA statement [32].

### Data sources and study eligibility

A comprehensive search of the scientific literature was conducted in PubMed, the Cochrane Central databases, the Commonwealth Agricultural Bureau, and Web of Science from inception through to September 2024 (Supplemental Table 2). The reference lists of any previously published systematic reviews on anthocyanins and health outcomes of interest were screened to identify additional potential studies. Search keywords and controlled vocabulary for anthocyanins (including constituents and anthocyanin-rich foods) and cardiometabolic health outcomes were based on a previous comprehensive search by Raman et al. [30] and refined with input from subject specialists AC and PC. A search filter to identify RCTs and prospective cohort studies was adapted from the Cochrane Highly Sensitive Search Strategy for identifying randomized trials (https://community.cochrane.org/search-filters). No start date or language restriction was applied. Duplicate results were removed using EndNote citation management software, and unique article records were uploaded to the web-based software Covidence for screening and data extraction (https://www.covidence.org/). Citations were screened in duplicate using the predefined eligibility criteria, and any disagreements regarding study inclusion or exclusion were resolved in group meetings, which included expert advice from AC and PC when necessary.

### Study inclusion/exclusion criteria

We included RCTs and prospective cohort studies conducted in adults (≥18 y) that quantified the amount of anthocyanins consumed per day or per week. For RCTs, studies that provided sufficient serving size data of anthocyanin-rich foods were also included when estimation of anthocyanin intake was possible using the USDA (https://agdatacommons.nal.usda.gov/articles/dataset/USDA_Database_for_the_Flavonoid_Content_of_Selected_Foods_Release_3_1_May_2014_/24659802) or Phenol Explorer databases (www.phenol-explorer.eu). This approach was novel, as previous systematic reviews have only included studies that reported anthocyanin intake. Interventions included anthocyanin supplements and foods and beverages rich in anthocyanins, such as berries, grapes, cherries, plums, red cabbage, and other fruits, vegetables, and grains that are naturally red, blue, or purple. Only RCTs in which the intervention group consumed ≥50 mg of anthocyanins per day were included. The justification for this approach was that it has previously been shown that intakes at this level are associated with meaningful CAD risk reduction [8]. The comparator arm included low or no anthocyanin content. Outcomes of interest included primary outcomes such as CVD clinical outcomes (e.g., CVD mortality, CAD, stroke, diabetes, and hypertension), and intermediate biomarkers of cardiometabolic health such as lipids and lipoproteins, BPs, anthropometrics, parameters associated with glycemic control (i.e., insulin, glucose, HbA1c, and HOMA-IR), endothelial function (i.e., FMD), arterial stiffness (i.e., PWV), and inflammation (i.e., hsCRP, IL-6, and TNF-α).

We excluded retrospective studies, cross-sectional studies, case series, case reports, and narrative reviews. Studies that had poorly defined interventions (for instance, in relation to the description of the anthocyanin-containing component) were excluded, as were anthocyanin studies in which the intervention contained alcohol (e.g., red wine).

We included prospective cohort studies that reported multivariable results adjusting for any potential confounders and had ≥6 mo of follow-up time. We only included RCTs that had ≥10 participants (healthy, overweight or obese) per intervention arm at baseline. Metabolic data were included when follow-up periods were ≥1 wk, whereas lipid and inflammatory marker data were included for studies with ≥3-wk follow-up (Supplemental Table 1).

### Data extraction

A standardized data extraction form was developed using the Covidence software application (https://www.covidence.org/). The form captured key study attributes, including study design, study population baseline characteristics, enrolled and analyzed sample size, intervention and comparators, outcomes of interest, results, and risk of bias assessment. Data from each study were independently extracted by 1 of 4 investigators and subsequently verified by ≥1 additional investigator. Any discrepancies were discussed, and if a consensus was not reached, disagreements were resolved in team meetings.

### Anthocyanin intake calculations

For RCTs that did not report quantities of anthocyanin consumption, but instead only reported amounts of intake of anthocyanin-rich foods, we calculated anthocyanin intakes using the USDA database (https://agdatacommons.nal.usda.gov/articles/dataset/USDA_Database_for_the_Flavonoid_Content_of_Selected_Foods_Release_3_1_May_2014_/24659802). The same procedure was followed to calculate intakes of the individual anthocyanins. When insufficient data were reported to calculate anthocyanin intake, we calculated intakes (mg/d) using product brochures or by contacting product manufacturers for additional anthocyanin data (which were previously not reported in the original publications).

### Risk of bias/quality assessment

Two reviewers independently assessed risk of bias for each of the included studies. For RCTs, we used the Cochrane Risk of Bias Tool for Clinical Trials, along with nutrition-specific criteria for critical appraisal of micronutrient systematic reviews [33,34]. Assessment included the risk of bias because of the randomization process, missing outcome data, and selection of reported results with an overall rating of low, some concern, or high. For prospective cohort studies, we used the Newcastle-Ottawa scoring system [35] rating each item with methodological quality as “Yes,” “No,” or “Unclear,” and with an overall quality of low, medium, or high.

### Data synthesis

All included studies were summarized both narratively and in summary tables, which reported the study design and outcomes of interest, and summarized key features of the study population, design, intervention, outcomes, and results.

We performed meta-analyses when ≥3 unique studies were deemed sufficiently similar in population and outcomes [36]. Studies with missing data that precluded analysis were excluded. Importantly, meta-analyses were analyzed separately for prospective cohort studies and RCTs, with RCT results converted to standardized units before analysis. For categorical outcomes, we used a random-effects model and reported the results as summary RRs, comparing the highest with lowest anthocyanin intake categories (as defined within each study). For continuous outcomes, we extracted mean baseline, final levels, and measures of variance (SD or SE) for both intervention and control groups. Within-cohort changes were calculated (final-baseline) for both intervention and control arms, with SEs estimated. Differences for continuous outcomes were calculated as:

net change = (intervention final − intervention initial) − (control final − control initial)

When ≥3 studies reported sufficient data, we subsequently conducted stratified analyses by *1*) anthocyanin type (i.e., cyanidin, delphinidin, malvidin, pelargonidin, peonidin, and petunidin), *2*) anthocyanin-rich food type (e.g., blueberries and cranberries), *3*) weeks of follow-up, and *4*) quartiles of total anthocyanin intake (i.e., dosage). When sufficient data were available, we also ran subgroup analyses by sex. For subanalyzes by “anthocyanin type,” we first calculated the percentage contribution of each anthocyanin type to the total anthocyanin content—an approach previously described in the analysis of anthocyanin structure on glucose and lipid metabolism [27]. In this study by Araki et al. [27], anthocyanin subtypes providing 50% total anthocyanin were analyzed; however, total anthocyanin intakes began at just 1.65 mg/d, and 7 included studies had total anthocyanin concentrations <50 mg/d. We performed meta-analyses for outcomes where a given anthocyanin type constituted ≥20% of the anthocyanin dose (in a sufficient number of studies)—given that we only included studies with total anthocyanin intakes ≥50 mg/d, this equated to an anthocyanin subtype intake of ≥10 mg/d.

To evaluate possible dose-response effects and heterogeneity in results, we considered metaregression analyses with continuous anthocyanin dosage (mg/d) and outcomes of interest. Because of the low number of studies by outcome, we used subgroup analyses to evaluate doses at different levels.

We tested between-study heterogeneity using the *Q* statistic (significant when *P* < 0.10) and quantified its extent with the *I*^2^ statistic [37]. *I*^2^ heterogeneity was classified as: >25% as low; >50% as moderate; and >75% as high. Statistical analyses were performed in Stata version 13 (StataCorp) using the metan, metareg, and metabias functions.

### Grading overall strength of evidence

A structured approach modeled using the Nutrition Evidence Systematic Review Methodology Manual [38] was used to assess the overall quality of evidence for each outcome as: Strong, Moderate, or Limited. The following criteria were evaluated for assessment: consistency, precision, quality, directness, and generalizability. A rating of Strong for Consistency was achieved for outcomes with strong consistency in direction and magnitude of the effect measured. Precision was determined by the size of the variance, as well as the studies being sufficiently powered. The quality measure was reflected by the overall quality of the studies assessed using the Newcastle-Ottawa Score [35] for each study. Directness was a measure of how direct the relationship was among intervention/exposure, comparator, and outcomes of primary interest in the systematic review question. Generalizability referred to the results being generalizable to the United States population of interest with regard to population characteristics, as well as the intervention under consideration and the outcomes that were reported. Additional details can be found in the supplementary material.

## Results

**FIGURE 1:**
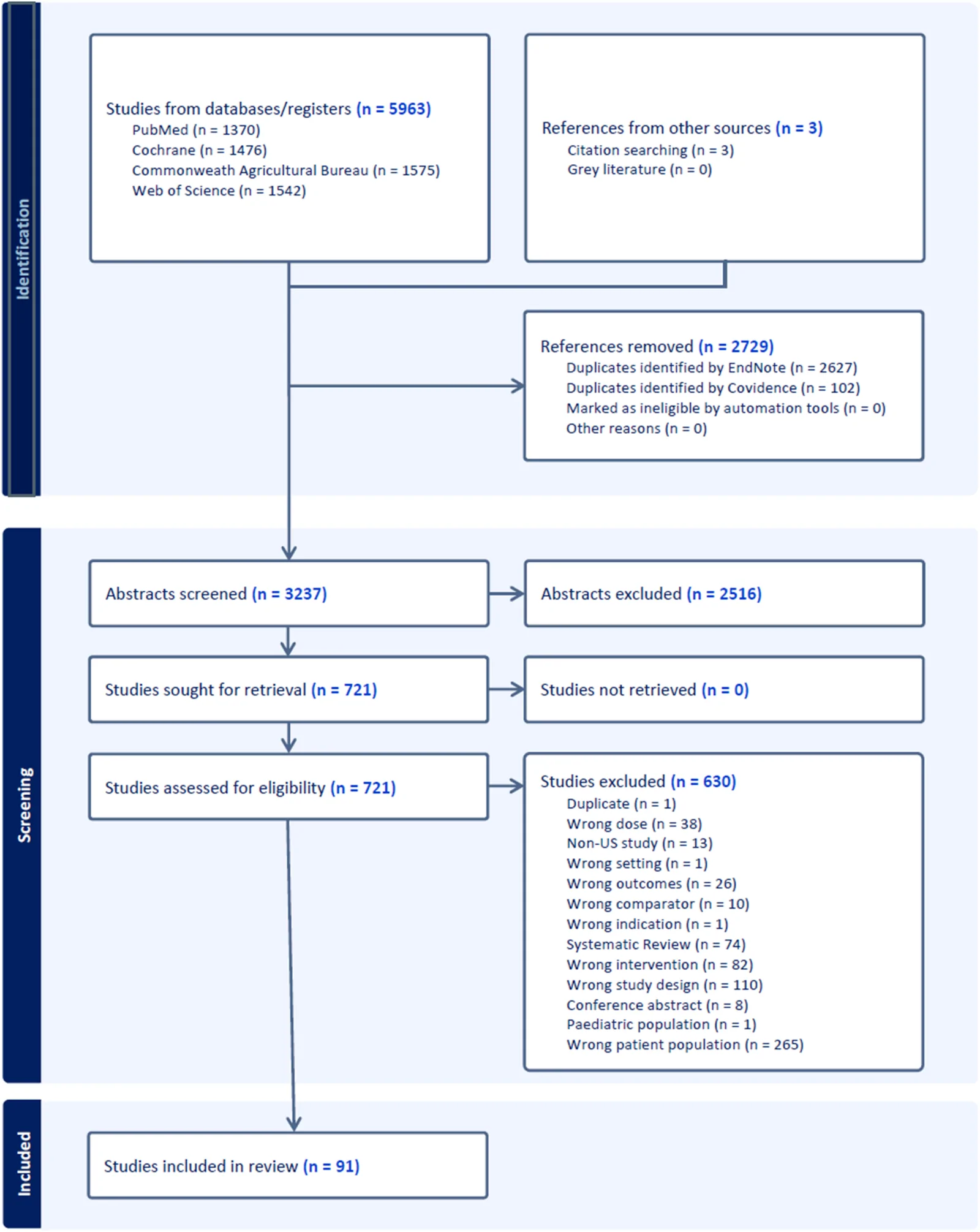
PRISMA study flow diagram of the review process. Abstracts identified (*n* = 5963); abstracts screened (*n* = 3237); abstracts not meeting criteria (*n* = 2516); full-text articles retrieved (*n* = 721); full-text articles excluded after screening (*n* = 630); full-text articles meeting study eligibility criteria (*n* = 91).

The database searches identified 3237 unique citations, of which 721 publications met the eligibility criteria and proceeded to full-text review (Figure 1). Of these, 18 prospective cohort studies in 27 publications and 65 RCTs met the inclusion criteria as depicted in the study flow diagram (Figure 1).

### Cohort studies

**FIGURE 2:**
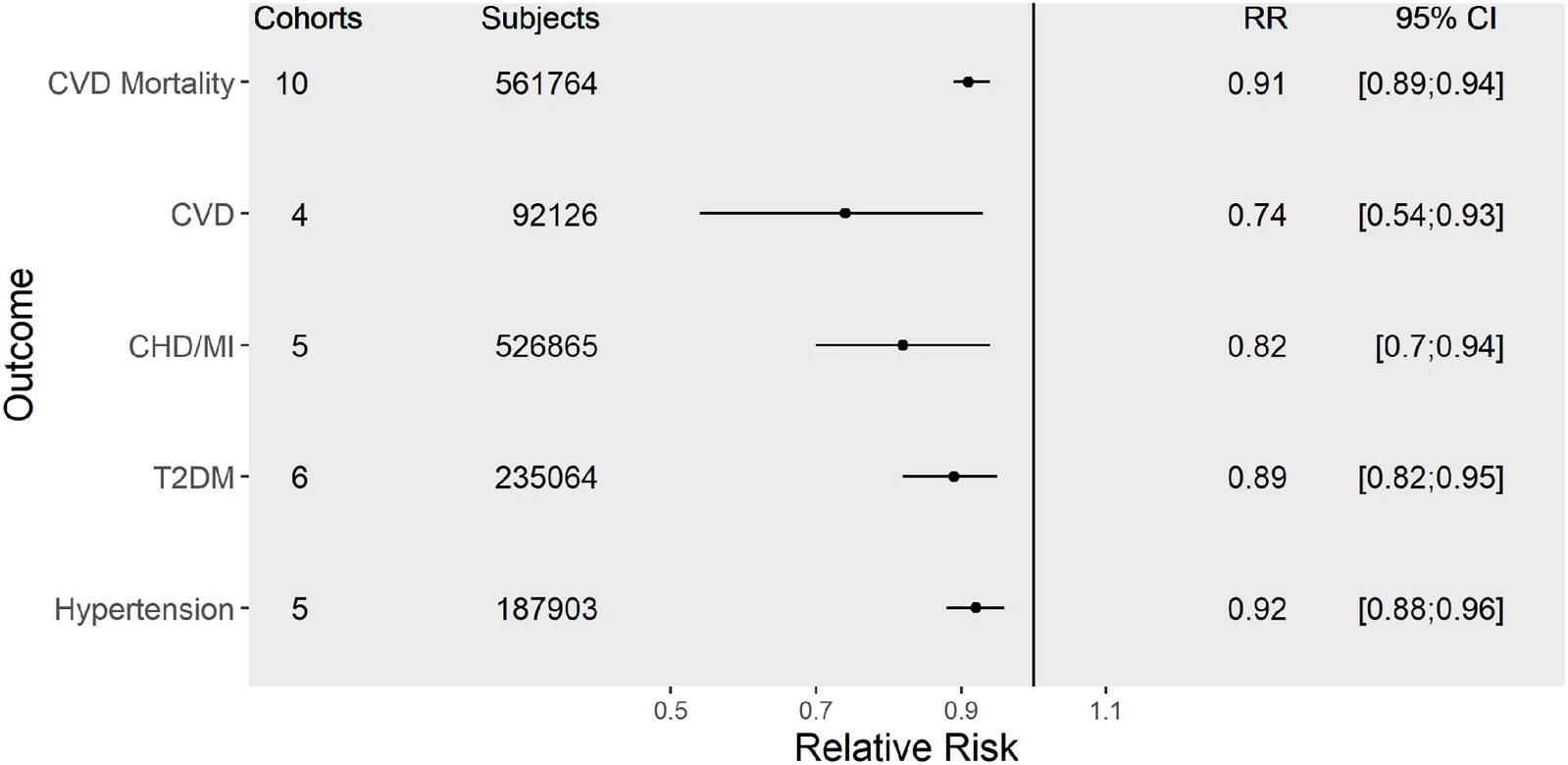
The association between anthocyanins and cardiometabolic health outcomes in prospective cohort studies. Data reported are relative risk and 95% CIs for a fully adjusted random-effects meta-analysis model for each outcome. ∗Hypertension outcome included male and female subpopulations from the same cohort from 1 article that reported anthocyanin intake and blood pressure. CI, confidence interval; CAD, chronic artery disease; CVD, cardiovascular disease; MI, myocardial infarction; T2DM, type 2 diabetes mellitus.

Eighteen cohort studies in 27 publications [6,7,[39], [40], [41], [42], [43], [44], [45], [46], [47], [48], [49], [50], [51], [52], [53], [54], [55], [56], [57], [58], [59], [60], [61], [62], [63]] met the inclusion criteria and reported the following outcomes: CVD mortality (*n* = 10) [6,7,41,42,51,52,57,60,62,63], incidence of CAD/myocardial infarction (MI) (*n* = 5) [39,43,44,48,53], stroke (*n* = 6) [7,39,43,46,47,56], hypertension (*n* = 3) [39,45,50,55], and T2DM (*n* = 6) [40,49,51,54,59,61] (Table 1). Participants were followed for between 4 and 24 y in these cohorts, and cohorts included between 1063 and 369,827 participants. Four cohorts included only female participants (Calcium Intake Fracture Outcome Age Related Extension Study, the European Prospective Investigation into Cancer and Nutrition, Nurses’ Health Study (NHS), NHS II), 1 included only males (Health Professional Follow-up Study), and the others included males at rates of between 14.7% and 51.8% of the cohort population. In these cohorts, anthocyanin intake was assessed by a semiquantitative food frequency questionnaire [5,6,[40], [41], [42], [43], [44], [45], [46], [47], [48], [49], [50], [51], [52], [53], [54], [55], [56], [57],^59^,[61], [62], [63], 24[5,6,40–57,59,61–63], 24-h dietary recall [39,60] 4-d food recording [7], and a beverage intake questionnaire [52]. Notably, a number of studies assessed anthocyanin intake with follow-ups ranging from 6 mo to 4 y [6,39,[42], [43], [44], [45], [46],^53^,^54^,^59^]. The models for all studies adjusted for participant characteristics such as age, BMI, and all but 2 cohorts adjusted for diet factors [52,55]. In addition, some studies adjusted for CVD risk factors, including T2DM or hypertension at baseline. The methodological quality of the studies demonstrated a low risk of bias, with 23 eligible publications rated as high quality and 4 as medium quality (Supplemental Table 3). A summary of the overall effects in meta-analyses for all eligible outcomes can be found in Figure 2 and Supplemental Table 4.

**TABLE 1:** Study characteristics of included cohorts

Author year, PMID	Country	Cohort name	Follow-up (y)	*n* enrolled (*n* analyzed)	% Male	Mean age (y)	BMI (kg/m^2^)	Funding source	Comorbidities (%)	Intake type, dose concentrations (mg/d)	Outcomes
Adriouch et al., 2022 30380657 [39]	France	NutriNet-Sante	8	84,158 (84,158)	21.3	44.1	NR	Fed; Ind	Diabetes, dyslipidemia, hypertension	27.5, 43.1, 55	CAD, CAD_MI, CVD, stroke
Adu et al., 2019 35380573 [40]	Australia	The Australian diabetes, obesity and lifestyle study	12	7675 (7675)	45	52	26.8	Fed; NP; Ind	CVD	Median: 12.6	Type 2 diabetes
Bondonno et al., 2019 31409784 [41]	Denmark	The Danish Diet Cancer and Health Cohort	23	56,048 (52,492)	47.6	56	25.5	Fed; NP	Diabetes, hypertension, hypercholesterolemia, CVD	5.2, 12.5, 20.0, 35.7, 70.7	CVD mortality
Bondonno et al., 2020 30718096 [42]	Australia	The Blue Mountains Eye Study	14	2349 (2349)	41.3	64.7	26	Fed	Hypertension, hypercholesterolemia	2.1, 5.6, 21.3	CVD mortality
Cassidy et al., 2016 21106916 [43]	United States	NHS, NHS II, HPFS	14	156,957 (156,957)	14.7	36	24.3	Fed	None reported	NHS: 6.8; 21.9 NHS II: 5.7, 16.2 HPFS: 6.7, 18	Hypertension
Cassidy et al., 2013 22363060 [44]	United States	NHS	14	69,622 (69,622)	0	NR	25.7	Fed	Diabetes, hypertension, hypercholesterolemia	5.8, 10.3, 13.6, 16.6, 16.5	Stroke
Cassidy et al., 2011 23319811 [45]	United States	NHS	18	93,600 (93,600)	0	36.6	24.6	Gov	Diabetes, hypertension, hypercholesterolemia	2.5, 5.0, 8.4, 13.5, 25.1	MI, CAD_MI
Cassidy et al., 2012 27488237 [46]	United States	HPFS	24	43,880 (43,880)	100	53.2	25.5	Fed	Diabetes, hypertension, hypercholesterolemia	1.9, 4.5, 7.8, 13.7, 26.3	MI, CAD_MI, stroke
Goetz et al., 2016 27655760 [47]	United States	REGARDS	6.5	30,239 (20,024)	44	64.6	29.1	Gov	Diabetes, hypertension	3.5, 6.3, 9.6, 14.6, 25.3	Stroke
Goetz et al., 2016 27655439 [48]	United States	REGARDS	6	16678 (16678)	41.2	64.3	29	Gov	None reported	4.0, 7.3, 10.3, 14.8, 24.7	CAD incidence
Grosso et al., 2017 28799519 [49]	Poland	HAPIEE	4	5806 (5806)	47	57.4	27.7	Fed; NP	None reported	4.4, 8.8, 14.5, 91.9	Type 2 diabetes
Grosso et al., 2018 [50]	Poland	HAPIEE	4	2725 (2725)	43	55.4	26.3	Fed; NP	None reported	Men: 4.4, 8.7, 14.4, 86.2 Women: 4.4, 8.8, 14.4, 106.2	Hypertension
Ivey et al., 2013 23628082 [52]	Australia	Calcium Intake Fracture Outcome Age related extension study	5	1063 (1063)	0	80.1	27.1	Gov, Fed	Diabetes, hypertension	<23, 23–41, >41	CVD mortality
Jacques et al., 2013 23902957 [54]	United States	Framingham Heart Study Offspring cohort	11.9	2915 (2915)	45.5	54.2	26.7	Gov, NP	Diabetes, CVD	2.5-fold increase in daily flavonoid intake	Type 2 diabetes
Jacques et al., 2015 26334117 [53]	United States	Framingham Offspring Heart Study	14.9	2880 (2880)	45.2	54.2	26.9	Fed; NP	Diabetes, bypertension, hypercholesterolemia	2.5-fold increase in daily flavonoid intake	CAD, CAD_MI, CVD
Lajous et al., 2016 26936332 [55]	French	EPIC	13.8	40,574 (40,574)	0	51.6	22.2	Gov, Fed	Diabetes, hypertension, hypercholesterolemia, CVD	39, 62, 75, 84, 94	Hypertension
McCullough et al., 2012 22218162 [6]	United States	American Cancer Society’s Cancer Prevention Study-II Nutrition Cohort	7	98,469 (98,469)	38.8	69.1	NR	Fed	Diabetes, hypertension, hypercholesterolemia, CVD	3.8, 6.8, 9.8, 13.7, 22.2	CVD mortality
Mursu et al., 2008 18377681 [7]	Finland	The Kuopio Ischemic Heart Disease Risk Factor Study	15.2	2682 (1950)	100	52.4	26.5	NP	Diabetes, CVD	Mean: 6.2	CVD mortality, stroke
Parmenter et al., 2021 37716608 [56]	Denmark	The Danish Diet, Cancer and Health Study	21	55,169 (55,169)	47.4	56	25.5	Fed; NP	Diabetes, hypertension, hypercholesterolemia, CVD	5, 13, 20, 36, 70	Stroke
Ponzo et al., 2015 [57]	Italy	NR	12	1658 (1658)	47	54.6	27	Fed	Diabetes, hypertension, hypercholesterolemia, CVD	Mean: 32.9	CVD, CVD mortality
Tresserra-Rimbau et al., 2014 24552647 [58]	Spain	PREDIMED	4.3	7172 (7172)	45.3	67.1	29.9	Fed	Diabetes, hypertension, hypercholesterolemia	11.8, 23.6, 32.8, 45.7, 74.6	CVD mortality
Tresserra-Rimbau et al., 2016 26962181 [51]	Spain	PREDIMED	4.3; 5.5	3430 (3430)	38.3	65.2	29.9	Fed; NP; Ind	Hypertension, hyperlipidemia	14.9, 30.9, 58.9	Diabetes
Wedick et al., 2012 22357723 [59]	United States	NHS, NHS II, HPFS	12–18	289,900 (199,980)	20.6	44.3	24.9	Gov	Diabetes, hypertension, hypercholesterolemia, CVD	NHS: 2.2, 4.7, 8.1, 13.1, 22.3; NHS II: 2.0, 4.5, 8.0, 13.7, 24.3, HPFS: 2.3, 4.9, 8.3, 14.0, 24.2	Type 2 diabetes
Yan et al., 2024 39496659 [60]	United States	NHANES	8	11,959 (11,959)	51.8	47.1	29.3	Fed	Diabetes, hypertension, hyperlipidemia	0.0, 0.9, 4.74, 28.0	CVD mortality
Zamora-Ros et al., 2013 24130345 [61]	Multiple	EPIC-InterAct	13.6	26,088 (15,258)	38	52.4	26	Aca, Fed, NP, Ind	Diabetes, hypertension, hyperlipidemia	7.1, 13.7, 20.6, 30.5, 51.6	Type 2 diabetes
Zamora-Ros et al., 2013 23881072 [62]	Spain	EPIC-Spain	13.6	41,438 (40,622)	38	49.3	NR	Fed	Diabetes, hypertension, hyperlipidemia	<5.8, 5.8–14.2, 14.2–22.2, 22.2–33.5, >33.5	CVD mortality
Zhao et al., 2023 36563462 [63]	United States	NIH-AARP Diet and Health Study	23.5	566,398 (369,827)	NR	61.2	NR	Fed; Ind	None reported	4.7, 8.7, 12.1, 16.5, 26.2	MI, CAD_MI, CVD mortality

Abbreviations: AARP, American Association of Retired Persons; CAD, coronary artery disease; CVD, cardiovascular disease; EPIC, European Prospective Investigation into Cancer and Nutrition; Fed, federal; Gov, government; HAPIEE, Health, Alcohol and Psychosocial factors in Eastern Europe; HPFS, Health Professionals Follow-up Study; Ind, industry; MI, myocardial infarction; NHS, Nurses’ Health Study; NP, nonprofit; NR, not reported; PREDIMED, Prevención con Dieta Mediterránea; REGARDS, REasons for Geographic and Racial Differences in Stroke; SES, socioeconomic status.

The association between a higher compared with lower habitual anthocyanin intake and CVD mortality was reported in 10 cohort studies from 10 publications [6,7,41,42,51,52,57,60,62,63], across 561,764 participants, who were followed-up over 6–24 y (Table 1). Ascertainment of CVD mortality was confirmed through national death indices or registries [6,7,41,42,52,[60], [61], [62], [63]], and death certificates [57]. A higher compared with lower habitual anthocyanin intake was associated with a 9% reduction in risk of CVD mortality [summary RR: 0.91; 95% confidence interval (CI): 0.89, 0.94; *I*^*2*^ = 14.7%; Supplemental Figure 1].

#### CVD

Four cohort studies in 4 publications [39,53,57,58] reported an association between a higher compared with lower habitual anthocyanin intake and risk of CVD across 92,126 participants, followed-up for 4–15 y (Table 1). Ascertainment of CVD was confirmed through self-report [39,58], physical exam, and study surveillance [53,57], review of medical records [39,53], and in the case of deceased participants, verification through national death indices [39,57]. Compared with low anthocyanin intakes, a higher compared with lower habitual anthocyanin intake was associated with a 26% reduction in risk of CVD (summary RR: 0.74; 95% CI: 0.54, 0.93; *I*^*2*^ = 80.6%; Supplemental Figure 2).

#### CHD/MI

Five cohorts (across 526,865 participants) in 5 publications [39,43,44,48,53], with a follow-up of 5–24 y, reported an association between a higher anthocyanin intake and CAD/nonfatal MI (Table 1). Ascertainment of CAD/MI was confirmed through self-reports [39,43], physical exams, and study surveillance [48,53], medical records [39,43,44,48,53], next-of-kin reports [48], and in the case of deceased participants, verification through national death indices [39,48] and autopsy reports [43,48]. A higher compared with lower habitual anthocyanin intake was associated with an 18% reduction in risk of CAD/MI in 5 cohorts (RR: 0.82; 95% CI: 0.70, 0.94; *I*^*2*^ = 53.6%; Supplemental Figure 3).

#### Type 2 diabetes

Eight cohorts from 6 publications [40,49,51,54,59,61] reported on the association between a higher compared with lower habitual intake of anthocyanins and risk of T2DM (across 235,064 participants, followed-up over 4–24 y) (Table 1). Ascertainment of T2DM was confirmed through self-reports [49,51,59,61], physical exams [40,54], medical records [59,61], and in the case of deceased participants, verification through national death indices and registries [61]. Higher compared with lower habitual anthocyanin intake was associated with an 11% reduction in the risk of developing T2DM (RR: 0.89; 95% CI: 0.82, 0.95; *I*^*2*^ = 46.9%; Supplemental Figure 4).

#### Hypertension

The association between higher compared with lower habitual anthocyanin intake and hypertension was reported in 5 cohorts from 3 publications [45,50,55] (across 182,097 participants, followed-up for 4–14 y) (Table 1). Ascertainment of hypertension was confirmed through self-reports [45] and physical exams [50], and in 1 case through verification from a health insurance plan’s drugs-claims database [55]. A higher compared with lower habitual anthocyanin intake was associated with an 8% reduction in risk of hypertension in 5 cohorts (summary RR: 0.92; 95% CI: 0.88, 0.96; *I*^*2*^ = 33.1%; Supplemental Figure 5). When we restricted meta-analysis to participants who were female (across *n* = 5 cohorts), a higher compared with lower habitual intake was associated with a 9% reduction in risk (RR: 0.91; 95% CI: 0.87, 0.94; *I*^*2*^ = 37.2%; Supplemental Figure 6).

#### Overall strength of evidence for cohort study outcomes

The overall strength of evidence for cohort study outcomes is shown in Supplemental Table 5. For the 5 outcomes for which meta-analysis was performed, evidence was graded as Moderate for incident CAD/nonfatal MI and CVD, whereas it was graded Limited for CVD mortality, T2DM, and hypertension. Detailed grading methodology in supplement.

### RCTs

**FIGURE 3:**
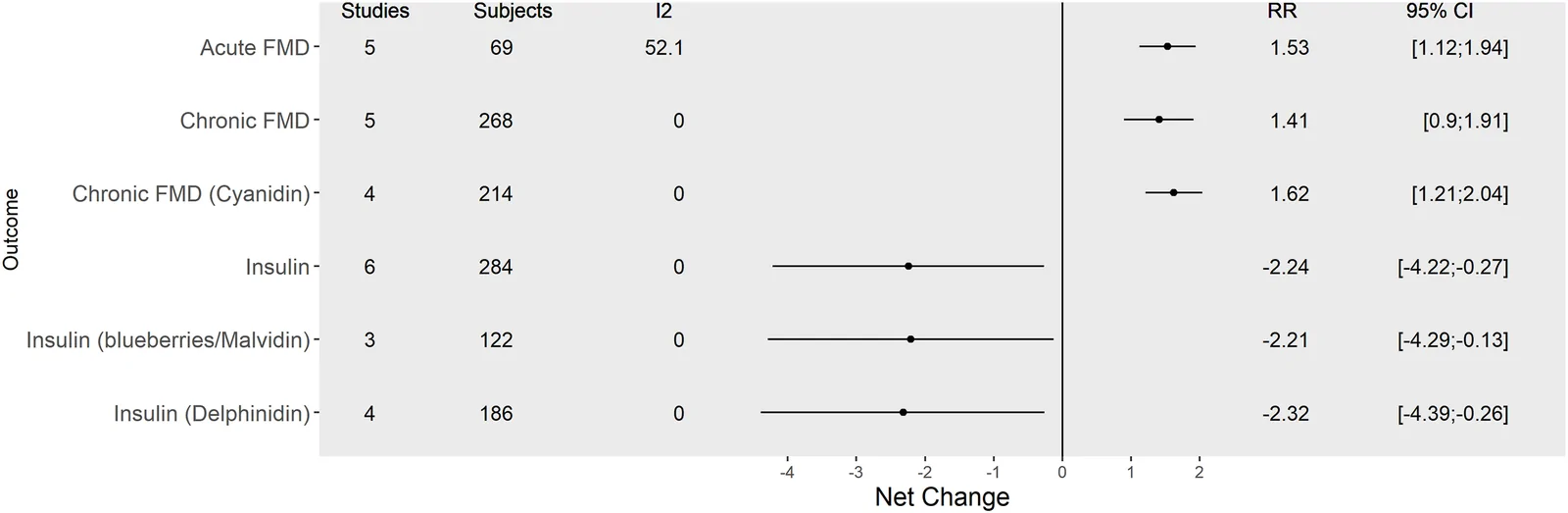
The effect of anthocyanin intake on cardiometabolic outcomes from RCTs. Net change and 95% CI are reported for each outcome. CI, confidence interval; FMD, flow-mediated dilation; RCT, randomized controlled trial; RR, relative risk.

A total of 65 RCTs met the inclusion criteria. Of these, anthocyanin intake was calculated for 50 studies that provided sufficient serving size data of anthocyanin-rich foods. Duration of the included studies ranged from interventions of 1 meal to 26 wk, although follow-up duration was <12 wk for the majority of studies. As expected, no RCT was of sufficient duration to evaluate hard clinical endpoints. Studies were conducted in 21 countries (most commonly, *n* = 16 from the United Kingdom and the United States, *n* = 6 from Germany, and *n* = 5 from Australia), and anthocyanin intakes ranged from 50 to 3300 mg/d, with a median intake of 211 mg/d. Studies included a median of 40 participants with the largest study including 130 participants. Descriptions of study characteristics and outcomes of interest are presented in Table 2. Sixteen RCTs were rated as having low risk of bias, 28 were rated as some concern, and 21 were rated as having a high risk of bias (Supplemental Table 6). A summary of the overall effects across the meta-analysis of all eligible outcomes is presented in Figure 3 and Supplemental Table 7.

**TABLE 2:** Baseline characteristics of randomized controlled trials

	Total [*n* (%)]	RCT parallel [*n* (%)]	RCT crossover [*n* (%)]
*n* publications	64 (100.00)	33 (51.6)	31 (48.4)
Mean age median (y)	38.5	44.2	38.0
Mean age	41.5	43.9	39.2
<50 y	42 (65.5)	18 (54.5)	24 (77.4)
≥50 y	20 (31.3)	13 (39.4)	6 (19.4)
Not reported	3 (4.7)	2 (6.0)	1 (3.2)
Mean analyzed *n* participants	40.4	48.2	22
Median analyzed *n* participants	36	35	20
Mean intervention duration (wk)	5.6	8.0	3
Median intervention duration (wk)	4	8	1
Intervention duration			
Acute	12 (18.8)	1 (3.0)	11 (35.4)
1 d to ≤1 wk	7 (10.9)	0	7 (22.5)
1 to ≤2 wk	2 (3.1)	1 (3.0)	1 (3.2)
2 to ≤4 wk	19 (29.7)	9 (27.3)	10 (32.3)
4 to ≤6 wk	6 (9.4)	6 (18.2)	0
>6 wk	19 (29.7)	17 (51.5)	2 (6.5)
Blinding			
Double-blind	41 (64.1)	20 (60.1)	21 (67.7)
Single-blind	8 (12.5)	3 (9.0)	5 (16.1)
Not blinded	1 (1.6)	1 (3.0)	0
Not reported	14 (22.1)	9 (27.3)	5 (16.1)
Study region			
Australia	5 (7.8)	0	5 (16.1)
Brazil	2 (3.1)	1 (3.0)	1 (3.2)
Canada	3 (4.7)	2 (6.1)	1 (3.2)
China	1 (1.5)	1 (3.0)	0
Costa Rica	1 (1.5)	0	1 (3.2)
Finland	1 (1.5)	1 (3.0)	0
France	0	0	0
Germany	6 (9.4)	4 (12.1)	2 (6.4)
India	1 (1.5)	1 (3.0)	0
Italy	2 (3.1)	1 (3.0)	1 (3.2)
Japan	1 (1.5)	0	1 (3.2)
Korea	1 (1.5)	1 (3.0)	0
Netherlands	1 (1.5)	0	1 (3.2)
New Zealand	1 (1.5)	0	1 (3.2)
Norway	2 (3.1)	2 (6.1)	0
Poland	1 (1.5)	1 (3.0)	0
Spain	0	0	0
Taiwan	1 (1.5)	0	1 (3.2)
Turkey	1 (1.5)	1 (3.0)	0
United Kingdom	16 (25.0)	8 (24.3)	8 (25.8)
United States	16 (25.0)	8 (24.3)	8 (25.8)
Intervention type			
Food	12 (18.8)	7 (21.2)	5 (16.1)
Beverage	36 (56.3)	16 (48.5)	20 (64.5)
Supplement	16 (25.0)	10 (30.3)	6 (19.3)
Intervention			
Acai	2 (3.1)	1 (3.0)	1 (3.2)
Pure anthocyanin	6 (9.4)	4 (12.1)	2 (6.4)
Aronia berry	1 (1.5)	1 (3.0)	0
Berry	1 (1.5)	1 (3.0)	0
Black chokeberry	1 (1.5)	0	1 (3.2)
Blackberries	2 (3.1)	0	2 (6.4)
Blackcurrant	5 (7.8)	2 (6.1)	3 (9.7)
Blood orange	1 (1.5)	0	1 (3.2)
Blueberries	13 (20.3)	7 (21.2)	5 (16.1)
Cherry	11 (17.2)	7 (21.2)	4 (12.9)
Cranberry	4 (6.3)	2 (6.1)	2 (6.4)
Elderberry	1 (1.5)	1 (3.0)	0
Grape	4 (6.3)	1 (3.0)	3 (9.7)
Maqui berry	1 (1.5)	1 (3.0)	0
Plum	3 (4.7)	0	3 (9.7)
Raspberry	3 (4.7)	2 (6.1)	1 (3.2)
Roselle	2 (3.1)	1 (3.0)	1 (3.2)
Strawberry	2 (3.1)	0	2 (6.4)
Mixed	1 (1.5)	1 (3.0)	0
Funding			
Academic	7 (10.9)	4 (12.1)	3 (9.7)
Federal	11 (17.2)	5 (15.2)	6 (19.4)
Industry	15 (23.4)	10 (30.3)	5 (16.1)
Nonprofit	1 (1.6)	0	1 (3.2)
Mixed	24 (37.5)	12 (36.4)	12 (37.5)
None	4 (6.3)	1 (3.0)	3 (38.7)
Not reported	2 (3.1)	1 (3.0)	1 (3.2)

Abbreviation: RCT, randomized controlled trial.

### Cardiovascular function and BP

#### Endothelial function

Acute postprandial FMD: meta-analysis of 7 RCTs [[64], [65], [66], [67], [68], [69]], which provided between 150 and 724 mg/d anthocyanin showed a significant increase in FMD, compared with the control group, when measured from half an hour to 3 h postprandially (net change: 1.40%; 95% CI: 1.11%, 1.69%; *I*^*2*^ *=* 6.0; Supplemental Figure 7); with moderate statistical heterogeneity. Furthermore, in subanalyzes by anthocyanin type, it was observed that malvidin and delphinidin intake increased postprandial FMD with low heterogeneity (net change: 1.69%; 95% CI: 1.20%, 2.18%; *I*^*2*^ = 0.0%; Supplemental Figures 8 and 9).

Chronic FMD: meta-analysis of 6 RCTs that reported FMD after anthocyanin intervention (ranging from 54 to 302 mg/d) for between 4 and 12 wk observed a significant FMD increase, with low heterogeneity (net change: 1.59%; 95% CI: 1.05%, 2.14%; *I*^*2*^ = 21.2%; Supplemental Figure 10). In addition, cyanidin [[70], [71], [72], [73]] and delphinidin [69,72,74] intake both significantly increased chronic FMD, with low heterogeneity (cyanidin net change: 1.62%; 95% CI: 1.21%, 2.04%; *I*^*2*^ = 0.0%; delphinidin net change: 1.59%; 95% CI: 1.05%, 2.14%; *I*^*2*^ = 21.2%; Supplemental Figure 11).

#### Blood pressure

Across 22 RCTs, we found no difference for diastolic or systolic BP after anthocyanin intervention (Supplemental Table 7).

### Markers of insulin and glycemic control

#### Insulin outcomes

Across 6 RCTs [73,[75], [76], [77], [78], [79]] which fed between 68 and 715 mg/d of anthocyanin for between 1 and 12 wk, a significant reduction in insulin concentration was observed compared with controls (net change: −2.24 pmol/L; 95% CI: −4.22, −0.27; *I*^*2*^ = 0.0%; Supplemental Figure 12). In the subanalysis by anthocyanin type, meta-analysis across 4 RCTs [75,[77], [78], [79]] confirmed a significant insulin decrease after delphinidin intakes over 1–8 wk (net change: −2.32 pmol/L; 95% CI: −4.39, −0.26, *I*^*2*^ = 0.0%; Supplemental Figure 13). Likewise, across 3 RCTs [[77], [78], [79]], lower insulin concentrations were also observed with higher malvidin intakes, with low heterogeneity (net change: −2.21 pmol/L; 95% CI: −4.29, −0.13; *I*^*2*^ = 0.0%; Supplemental Figure 14). The same 3 studies also represent our subgroup analysis for blueberries. Other anthocyanins were not analyzed for reasons outlined in the Methods section.

#### Other glycemic biomarkers

Meta-analysis of RCTs reporting on glucose, HbA1c, and HOMA-IR found no difference between anthocyanin intake and controls (Supplemental Table 7).

### Anthropometric measurements, arterial stiffness, lipids, lipoproteins, and biomarkers of inflammation

Meta-analyses of RCTs reporting on BMI, body weight, % body fat, waist circumference, IL-6, hsCRP, oxidized LDL, PWV, TNF-α, apolipoprotein A1, apolipoprotein B, total cholesterol, LDL cholesterol, HDL cholesterol, and triglycerides observed no difference after anthocyanin intervention. However, when subanalyzed by anthocyanin type, a higher delphinidin intake resulted in significant reductions in chronic PWV (net change: −0.33; 95% CI: −0.62, −0.05; *I*^*2*^ = 0.0%; Supplemental Figure 15) and triglyceride concentrations (net change: −0.10 mmol/L; 95% CI: −0.17, −0.03; *I*^*2*^ = 0.0%; Supplemental Figure 16).

#### Sensitivity analyses

We performed the same sensitivity analyses for outcomes subanalyzed by “food type,” and in this case, blueberry was the only food to reach the threshold criteria, and only insulin was significantly decreased after blueberry intake (net change: −2.21 mmol/L; 95% CI: −4.29, −0.13; *I*^*2*^ = 0.0%; Supplemental Figure 14). Notably, we also observed no anthocyanin dose-response effect for any measure.

Sensitivity analyses were also performed for outcomes where the risk of bias for RCTs was low (i.e., good methodology, and where ≥3 included studies were of low risk of bias for a given outcome). In meta-analyses across only studies which met this threshold, and for the outcomes which follow, no difference was observed between intervention and control for: BMI, body fat, body weight, diastolic blood pressure (DBP), glucose, HbA1c, HOMA-IR, HDL cholesterol, hsCRP, LDL cholesterol, TNF-α, systolic blood pressure (SBP), total cholesterol, triglycerides, and waist circumference. For FMD, all included studies had low risk of bias, and therefore, no separate sensitivity analyses were required.

#### Overall strength of the evidence for RCTs

Supplemental Table 8 shows the grading of the strength of evidence of the outcomes reported from the RCTs. Of the outcomes for which meta-analysis was performed, the evidence was graded as strong for acute FMD and moderate for chronic FMD. For insulin, the lack of a consistent effect relative to the zero line, as well as the wide variance, resulted in an assessment as limited for the strength of evidence.

### Discussion

To our knowledge, this is the first systematic review and meta-analysis to integrate evidence from both prospective cohort studies and RCTs involving individuals who are healthy/disease-free to evaluate the preventive effects of anthocyanin intake on intermediate cardiometabolic biomarkers and long-term cardiovascular (CV) health outcomes. Across 18 prospective cohort studies, higher habitual anthocyanin intake was associated with a risk reduction of 8%–26% in CVD mortality, CVD incidence, MI, hypertension, and T2DM. The evidence for CVD/MI incidence was of moderate strength based on Grades of Recommendation, Assessment, Development, and Evaluation (GRADE).

Our meta-analysis of 65 RCTs conducted in healthy populations, receiving ≥50 mg/d anthocyanin, provided mechanistic support for these findings. Anthocyanins improved endothelial function both postprandially (1.53%) and chronically (1.41%)—the latter aligning with a 17% reduction in CV events based on prognostic data for FMD [80] and compares favorably with prior meta-analyses of heterogeneous populations (i.e., 2 healthy study populations, a smoking population, and populations with metabolic syndrome, CAD, elevated CVD risk factors, prehypertension, and hypercholesterolemia) reporting a smaller 0.84% increase [24]. The magnitude was also comparable to our flavan-3-ol meta-analysis (1.21%, *n* = 23 trials) [30], suggesting a mechanistic overlap with both flavonoid subclasses improving endothelial function. Arterial stiffness also improved postprandially (PWV −0.24 m/s), consistent with prior meta-analyses involving individuals with varied health statuses [24]. Importantly, higher anthocyanin doses (≥200 mg) consumed for ≥1 wk reduced chronic arterial stiffness (PWV −0.27 m/s)—a novel finding not previously reported [24]. Notably, GRADE analyses indicated strong evidence for the acute FMD effects, moderate evidence for chronic FMD and acute PWV, but we found no significant effect of anthocyanins on BP.

The absence of a BP effect in healthy participants was unexpected, given improvements in FMD and PWV. However, this aligns with our findings from a 6-mo RCT in participants with metabolic syndrome, where clinically relevant improvements in FMD and PWV were observed, without BP changes [81]. Notably, trials using 24-h ambulatory BP (ABP) reported reductions after blueberry or raspberry intake [69,74]. Office BP may mask subtle regulatory changes compared with 24-h ABP [82], which more accurately predicts mortality [83,84] and risk features derived from segmented periods, e.g., BP dipping, elevated night-to-day ratio, high night SBP, isolated nocturnal masked hypertension, and both daytime and nocturnal masked hypertension [85,86]. Where feasible, future trials should standardize ABP protocols by defining daytime and nocturnal periods, ensuring adequate readings during sleep, and applying quality checks to include only protocol-compliant data. When ABP is impractical, repeated morning and evening home BP measurements provide a superior alternative to single office readings.

Anthocyanin intake also reduced fasting insulin (−2.24 pmol/L insulin), with similar effects for blueberries (−2.21 pmol/L insulin), delphinidin-rich (−2.32 pmol/L insulin), and malvidin-rich (−2.21 pmol/L insulin) foods. Although other glycemic biomarkers were unchanged in healthy participants (e.g., fasting blood glucose, postprandial 2 h blood glucose, HbA1c, and HOMA-IR), these insulin effects may reflect improved insulin sensitivity under normoglycemic conditions. Consistent with this, our meta-analysis across 6 prospective cohort studies [40,49,51,54,59,61] showed an 11% reduction in T2DM risk with higher anthocyanin intake—marginally lower than the 15% reduction reported across 3 prospective cohorts in a 2012 umbrella review [11]. Together, these findings support a role for anthocyanins in maintaining glucose homeostasis during good health, with greater effects on glycemic endpoints emerging in populations with existing metabolic dysfunction [16,13,15].

Across other cardiometabolic risk biomarkers (e.g., hsCRP, TNF-α, and triglycerides), effects were nonsignificant and small, likely reflecting physiological stability in healthy populations. Composite biomarker panels may better capture subtle multisystem effects in future research with such healthy populations.

A novel element of this review was the differentiation by anthocyanin type when assessing cardiometabolic health effects. In addition to the previously noted benefits of delphinidin on insulin concentrations, this anthocyanin also reduced arterial stiffness (PWV −0.33 m/s) and triglycerides (–0.10 mmol/L). Similarly, cyanidin improved FMD (+1.62%). These findings suggest that differences in anthocyanin chemical structure may influence biological activity, and given that foods provide mixed profiles of anthocyanin types, such combinations may act across multiple mechanisms that collectively reduce the risk of CVD and T2DM. Previous findings support that anthocyanins mediate vascular health benefits via modulation of nitric oxide synthase, platelet aggregation, and cyclooxygenase-2 expression [87], and cyanidin has been shown to have hypotensive effects, mediated by rapid penetration into endothelial cells (with rapid elimination from plasma), which may explain its FMD benefits [[88], [89], [90]]. Likewise, delphinidin-mediated cardio-protection has been reported via adenosine monophosphate-activated protein kinase/NADPH oxidase/mitogen-activated protein kinase signaling [91].

Compared with earlier reviews [10,12,23], our analysis included substantially more RCTs because of the use of a robust method for estimating anthocyanin content when it was not directly quantified in intervention foods. Reassuringly, our findings are consistent with those of the more limited prior reviews [10,12,23]. Nonetheless, limitations remain. Study quality varied considerably, with 48% of trials assessing BP and 46% of trials assessing HDL classified as low quality, introducing a high risk of bias. In our previous meta-analysis of flavan-3-ols, we included 15 prospective cohort studies and 156 RCTs [30], whereas the current review included a similar number of cohort datasets (*n* = 18) but only 65 RCTs. This inevitably limited our ability to conduct sensitivity analyses exploring the effects of dietary form, dose, and duration on the cardiometabolic biomarkers. In the cohort studies, anthocyanin intakes were estimated using food composition databases applied to self-reported dietary data—a method that distinguishes between broad intake categories but cannot capture the extensive metabolism these compounds undergo postingestion. Future cohort studies should, therefore, integrate biomarker-based assessments to better characterize exposure to anthocyanins and their metabolites on CV health. Moreover, the use of the highest anthocyanin intake category from each cohort may introduce bias, where some studies report substantially greater anthocyanin intakes than others. For RCTs, many interventions did not independently quantify anthocyanin content; we addressed this by applying a standardized estimation method in 50 RCTs. Additional limitations include the small average sample size of RCTs and the presence of other flavonoids and phenolic acids with anthocyanin-rich foods, which may contribute, individually or synergistically, toward the cardiometabolic effects observed. Although heterogeneity was generally low in both cohort and RCT meta-analyses, considerable heterogeneity was observed in CVD incidence, likely due to differences in anthocyanin intake and follow-up duration. Despite these limitations, by focusing on only high-quality studies with meaningful anthocyanin doses (≥50 mg/d) and applying GRADE, we were able to conduct robust meta-analyses and identify key research gaps in the field.

By integrating all available human data from high-quality prospective cohort studies and RCTs, our findings suggest that increased intake of anthocyanin-rich foods is associated with cardiometabolic health benefits in individuals and populations who were healthy and disease-free. Most notably, the observed +1.41% improvement in FMD, a marker of chronic endothelial function, may have meaningful implications for CVD risk. This, alongside modest yet robust improvements in arterial stiffness (i.e., PWV) and insulin concentrations could translate into significant long-term health benefits. Larger and longer-duration trials are now needed, particularly those that can disentangle the specific effects of anthocyanins from other bioactive constituents in foods. Future research should prioritize biomarker-based intake assessments in cohort studies and explore dose-response relationships in RCTs. Collectively, such data will be essential for informing and refining evidence-based dietary guidelines for CVD prevention.

## Author contributions

The authors’ responsibilities were as follows — NN, PC, AC: conceived and designed the study; EEA, ED, HE, AVN, LC, MW, SHS, SM: performed the data extraction and quality assessment; EEA, NN, ED, HE: synthesized and analyzed the data; EEA, PC, NN, AC: interpreted and further analyzed the data; PC, NN, EEA, ED, HE, AC: wrote the paper; NN: had primary responsibility for final content; and all authors: read and approved the final manuscript.

## Data availability

Data described in the manuscript, code book, and analytic code will be made available on request pending approval.

## Declaration of generative AI and AI-assisted technologies in the writing process

The author(s) declare that no generative AI or AI-assisted technologies were used in the writing of this manuscript.

## Funding

This research was in part supported by the US Highbush Blueberry Council (USHBC, with oversight from the USDA). The funder played no role in the study selection, quality assessment, data synthesis, or manuscript preparation. AC is Co-Director of the Co-Centre for Sustainable Food Systems [funded by Research Ireland, Northern Ireland’s Department of Agriculture, Environment and Rural Affairs (DAERA), and UK Research and Innovation (UKRI) via the International Science Partnerships Fund (ISPF), under Grant number 22/CC/11147].

## Conflict of interest

This research was in part supported by the US Highbush Blueberry Council (USHBC, with oversight from the US Dept. of Agriculture). The funder played no role in the study selection, quality assessment, data synthesis, or manuscript preparation. AC acts as an advisor to the USHBC grant committee and has received funding from them for a randomized controlled blueberry trial, as well as population-based work. All other co-authors had no other conflict of interest to report.
