# Supplementary material for: Dietary intakes of anthocyanins and cardiometabolic health: a systematic review and meta-analysis of randomized trials and prospective cohort studies in healthy participants
Source: Am J Clin Nutr. 2026 Jun 18;124(2):101304. doi: 10.1016/j.ajcnut.2026.101304 (PMC13494092; doi:10.1016/j.ajcnut.2026.101304)
Supplement: Supplementary file 1 [file mmc1.docx]

**Dietary intakes of anthocyanins and cardiometabolic health: A systematic review and meta-analysis of randomized trials and prospective cohort studies in healthy participants**

Avendano et al.

**Supplemental Materials**

Table of Contents

[**Supplemental Table 1:** Inclusion/Exclusion criteria	3](#_Toc221114166)

[**Supplemental Table 2:** Pubmed Search strategy	5](#_Toc221114167)

[**Text 1:** Details of the Grading protocol applied in this analysis	7](#_Toc221114168)

[**Supplemental Table 3:** Quality of included prospective cohort studies	8](#_Toc221114169)

[**Supplemental Table 4.** Results from prospective cohort studies examining anthocyanin	10](#_Toc221114170)

[**Supplemental Figure 1.** The effect of anthocyanins on CVD mortality in prospective cohort studies	11](#_Toc221114171)

[**Supplemental Figure 2.** The effect of anthocyanins on CVD in prospective cohort studies	11](#_Toc221114172)

[**Supplemental Figure 3.** The effect of anthocyanins on CHD+MI in prospective cohort studies	11](#_Toc221114173)

[**Supplemental Figure 4.** The effect of anthocyanins on Type 2 Diabetes in prospective cohort studies	12](#_Toc221114174)

[**Supplemental Figure 5.** The effect of anthocyanins on Hypertension in prospective cohort studies	12](#_Toc221114175)

[**Supplemental Figure 6.** Subgroup analysis of female cohorts on the effect of anthocyanins on Hypertension in prospective cohort studies	12](#_Toc221114176)

[**Supplemental Table 5.** GRADE overall quality for outcomes from prospective cohort studies	13](#_Toc221114177)

[**Supplemental Table 6:** Risk of bias assessment of Randomized Controlled Trials	14](#_Toc221114178)

[**Supplemental Table 7.** Results from anthocyanin randomized controlled trials meta-analyses	18](#_Toc221114179)

[**Supplemental Figure 7.** The effect of anthocyanins on acute FMD in randomized controlled trials	24](#_Toc221114180)

[**Supplemental Figure 8.** The effect of Malvidin on acute FMD in randomized controlled trials	24](#_Toc221114181)

[**Supplemental Figure 9.** The effect of Delphinidin on acute FMD in randomized controlled trials	25](#_Toc221114182)

[**Supplemental Figure 10.** The effect of anthocyanin on chronic FMD in randomized controlled trials	25](#_Toc221114183)

[**Supplemental Figure 11.** The effect of Delphinidin on chronic FMD in randomized controlled trials	26](#_Toc221114184)

[**Supplemental Figure 12.** The effect of anthocyanin on chronic Insulin in randomized controlled trials	26](#_Toc221114185)

[**Supplemental Figure 13.** The effect of Delphinidin on insulin in randomized controlled trials	26](#_Toc221114186)

[**Supplemental Figure 14.** Subgroup analysis of the effect of anthocyanin in blueberries/malvidin on Insulin in randomized controlled trials.	27](#_Toc221114187)

[**Supplemental Figure 15.** The effect of Delphinidin on chronic PWV in randomized controlled trials	27](#_Toc221114188)

[**Supplemental Figure 16.** The effect of Delphinidin on triglycerides in randomized controlled trials	27](#_Toc221114189)

[**Supplemental Table 8.** GRADE overall quality for outcomes from randomized controlled trials	28](#_Toc221114190)

# **Supplemental Table 1:** Inclusion/Exclusion criteria

| **Inclusion** | **Exclusion** |
| --- | --- |
| **Population** | |
| Adults (age≥18) | Non-human subjects (e.g., in vitro, cell, stem cell, or animal studies) |
| Healthy | Any children and adolescents (< 18 years old) |
| Baseline obese or overweight | Pregnant or lactating females |
| Blood pressure <140/90 mmHg;  Cholesterol <200 mg/dl (5.172 mmol/l) | Adults with chronic, inflammatory, or autoimmune disease conditions (e.g., rheumatoid arthritis, polycystic ovarian syndrome, chronic kidney disease, organ transplant) |
|  | Non-healthy subject (e.g., obstructive sleep apnea, cognitive disorders, etc.) |
|  | Adults with increased risk of CVD (e.g., prehypertension, hypertension, diabetes, metabolic syndrome, etc.) |
|  | Adults with CVD |
|  | 100% smokers |
|  | Participants in concurrent cancer or exercise trials |
| **Intervention** | |
| Red, blue, or purple fruits and vegetables that are fresh, frozen, or dried, or other food products that contain anthocyanins. (include blueberry leaves) | No red, blue, or purple food |
| Anthocyanins supplement, extract, or pure compound (e.g., cyanidin, delphinidin, petunidin, peonidin, pelargonidin, and malvidin) | Foods with no anthocyanin or amounts below threshold of significance (for RCTs, <50 mg/day, for cohorts, <5 mg/day) |
|  | Interventions including alcohol |
| **Comparator** | |
| No anthocyanins | Red, blue, or purple fruits and vegetables |
| Foods with no anthocyanin content | Anthocyanins supplement, extract, or pure compound |
| **Outcome** | |
| Cardiovascular disease (CVD) clinical outcomes  (≥ 6 months follow-up), including acute coronary syndrome, angina, arteriosclerosis, atherosclerosis, atrial fibrillation, cerebrovascular disorders, coronary artery disease, heart failure, myocardial infarction, peripheral vascular disease, and venous thromboembolism |  |
| Serum lipids (≥ 3 weeks follow-up), including blood pressure (BP), diastolic BP (DBP), low-density lipoprotein (LDL), high-density lipoprotein (HDL), systolic BP (SBP), total cholesterol, triglycerides |  |
| Metabolic parameters (≥ 1 week follow-up), including blood glucose, hemoglobin A1C (HbA1C), homeostatic model assessment and insulin resistance (HOMA-IR), insulin resistance, quantitative insulin sensitivity check index (QUICKI) |  |
| Inflammatory markers (≥ 3 weeks follow-up), including high-sensitivity C-reactive protein (hs-CRP), intercellular adhesion molecule 1 (ICAM-1), interleukins, vascular cell adhesion molecule 1 (VCAM-1) |  |
| Evaluation of endothelial function (any study length, including acute studies), using Endo-PAT, flow-mediated dilation (FMD), pulse-amplitude, pulse wave analysis, pulse wave velocity |  |
| **Design** | |
| RCTs | Cross-sectional studies |
| Prospective cohort studies | Retrospective cohort studies |
|  | Case reports |
|  | Case series |
|  | Reviews, lectures, opinion articles, news articles, proposed studies (i.e., no results reported) |
|  | Systematic Reviews |
|  | Studies with < 10 subjects per arm of interest |
|  | Studies where drugs are being tested |

# **Supplemental Table 2:** Pubmed Search strategy

| **Search number** | **Query** |
| --- | --- |
| 1 | "Anthocyanins"[Mesh] OR "Vaccinium"[Mesh] OR "Rubus"[Mesh] OR "Prunus domestica"[Mesh] OR "Vitis"[Mesh] OR "Prunus avium"[Mesh] OR "Wine"[Mesh] OR "Sambucus"[Mesh] OR "Ribes"[Mesh] OR "Brassica"[Mesh] OR "Onions"[Mesh] OR "Raphanus"[Mesh] OR "Photinia"[Mesh] OR "Euterpe"[Mesh] OR "Fragaria"[Mesh] OR "Solanum melongena"[Mesh] OR "Phaseolus"[Mesh] OR "Solanum tuberosum"[Mesh] |
| 2 | Anthocyanin* [tiab] OR cyanidin* [tiab] OR delphinidin* [tiab] OR petunidin* [tiab] OR peonidin* [tiab] OR pelargonidin* [tiab] OR malvidin* [tiab] OR blueberr* [tiab] OR blackberr* [tiab] OR "black plum*" [tiab] OR raspberr* [tiab] OR "Concord grape*" [tiab] OR "red grape*" [tiab] OR cherr* [tiab] OR "red wine*" [tiab] OR chokeberr* [tiab] OR elderberr* [tiab] OR currant* [tiab] OR "red cabbage*" [tiab] OR "red onion*" [tiab] OR "red radish*" [tiab] OR acai* OR billberr* [tiab] OR whortleberr* [tiab] OR cranberr* [tiab] OR lingonberr* [tiab] OR maqui* [tiab] OR strawberr* [tiab] OR eggplant* [tiab] OR "black bean*" [tiab] OR "purple potato*" [tiab] OR "purple wheat*" [tiab] |
| 3 | #1 OR #2 |
| 4 | "Cardiovascular Diseases"[Mesh] OR "Diabetes Mellitus"[Mesh] OR "Blood Glucose"[Mesh] OR "Lipoproteins, LDL"[Mesh] OR "Insulin Resistance"[Mesh] OR "C-Reactive Protein"[Mesh] OR "Interleukin-6"[Mesh] OR "Interleukin-1beta"[Mesh] OR "Pulse Wave Analysis"[Mesh] OR "NF-kappa B"[Mesh] OR "Cell Adhesion Molecules"[Mesh] OR "Tumor Necrosis Factor-alpha"[Mesh] OR "Leptin"[Mesh] OR "Isoprostanes"[Mesh] OR "Glycated Hemoglobin"[Mesh] OR "Hyperlipidemias"[Mesh] OR "Cholesterol"[Mesh] OR "Triglycerides"[Mesh] |
| 5 | cardiovascular disease* [tiab] OR heart disease* [tiab] OR myocardial infarction* [tiab] OR stroke* [tiab] OR cerebrovascular disorder* [tiab] OR coronary artery disease* [tiab] OR coronary heart disease* [tiab] OR heart attack* [tiab] OR sudden death [tiab] OR arteriosclerosis [tiab] OR atherosclerosis [tiab] OR heart failure* [tiab] peripheral vascular disease* [tiab] OR blood pressure [tiab] OR hypertension [tiab] OR diabet* [tiab] OR blood glucose [tiab] OR vascular disease* [tiab] OR atrial fibrillation [tiab] OR oxidized low density lipoprotein [tiab] OR insulin resistance [tiab] OR hsCRP [tiab] OR high sensitivity C-reactive protein [tiab] OR interleukin-6 [tiab] OR interleukin-1b* [tiab] OR pulse wave analysis [tiab] OR flow mediated dilation [tiab] OR endotheli* [tiab] OR acute coronary syndrome [tiab] OR angina pectoris [tiab] OR venous thromboembolism [tiab] OR venous thrombosis [tiab] OR insulin resistance [tiab] OR NF-kappa B [tiab] OR intercellular adhesion module* [tiab] OR tumor necrosis factor-alpha [tiab] OR leptin [tiab] OR isoprostane* [tiab] OR glycosylated hemoglobin [tiab] OR hyperlipidemia* [tiab] OR hypercholesterolemia* [tiab] OR cholesterol* [tiab] OR triglyceride* [tiab] OR prediabet* [tiab] |
| 6 | #4 OR #5 |
| 7 | "Clinical Trials as Topic"[Mesh] OR "Prospective Studies"[Mesh] OR "Random Allocation"[Mesh] OR "Double-Blind Method"[Mesh] OR "Single-Blind Method"[Mesh] |
| 8 | "Clinical Trial" [Publication Type] |
| 9 | randomized controlled trial* [tiab] OR RCT [tiab] OR controlled clinical trial* [tiab] OR clinical trial* [tiab] OR prospective stud* [tiab] OR cross-over stud* [tiab] OR random allocation [tiab] OR placebo* [tiab] OR random* [tiab] OR trial* [tiab] OR cohort* [tiab] OR parallel stud* [tiab] |
| 10 | #7 OR #8 OR #9 |
| 11 | #3 AND #6 AND #10 |
| 12 | "Animals"[Mesh] NOT "Humans"[Mesh] |
| 13 | #11 NOT #12 |

# **Text 1:** Details of the Grading protocol applied in this analysis

- *Grading approach for Cohort studies:* All outcomes were graded as “strong” for the Quality element since the Newcastle-Ottawa score was either High or Medium. All outcomes were graded as “not assignable” for directness since typically in nutrition-related studies the extent to which studies are designed to directly examine the relationship between the interventions/exposures, comparators, and outcomes may not be unequivocal. For the generalizability element, since studies were conducted either in the US or in countries with primarily white populations, outcomes were graded as moderate, except for T2DM, which showed a strong generalizability due to the cohorts in the meta-analysis being largely based in the US. The overall grade, therefore, was primarily influenced by the precision and consistency elements in the grading rubric.
- *Grading approach for RCTs*: For the Quality element, the overall risk of bias was graded to be “Strong” if the majority of the studies had a low risk of bias, “Moderate” if the studies had no more than one element with a high risk of bias, and all other studies were low or “some concern” risk of bias, and “Limited” if the majority of studies had a high risk of bias. For directness, the grade was “Strong” if all the studies had the same food type or if all the studies had anthocyanin supplements, “Moderate” for all other studies. For the generalizability element, since studies were conducted either in the US or in countries with primarily white populations, outcomes were graded as moderate. The grading for Consistency and Precision is elaborated for each outcome in supplementary text. For directness, we graded the element according to the diversity of interventions reported in the studies included for the meta-analysis.

# **Supplemental Table 3:** Quality of included prospective cohort studies

| **Author Year** | **Selection** | | | | **Comparability of cohorts on the basis of the design or analysis** | **Outcomes** | | | **Total * for Outcome** |
| --- | --- | --- | --- | --- | --- | --- | --- | --- | --- |
|  | **Representativeness of the exposed cohort** | **Selection of the non-exposed cohort** | **Ascertainment of exposure** | **Demonstration that outcome of interest was not present at start of study** |  | **Assessment of outcome** | **Follow-up long enough for outcomes to occur** | **Adequacy of follow up of cohorts** |  |
| Adriouch 2018 | * | * | * | * | ** | * | * |  | ******** |
| Adu 2022 | * | * | * | * | ** | * | * | * | ********* |
| Bondonno 2019 | * | * |  | * | ** | * | * | * | ******** |
| Bondonno 2020 | * | * |  | * | ** | * | * | * | ******** |
| Cassidy 2011 |  | * | * | * | * |  | * |  | ***** |
| Cassidy 2012 |  | * |  | * | ** | * | * | * | ******* |
| Cassidy 2013 |  | * | * | * | ** | * | * |  | ******* |
| Cassidy 2016 |  | * | * | * | ** | * | * |  | ******* |
| Goetz 2016 | * | * |  | * | ** | * |  | * | ******* |
| Goetz 2016 | * | * |  | * | ** |  |  | * | ****** |
| Grosso 2017 | * | * | * | * | ** |  | * |  | ******* |
| Grosso 2018 | * | * |  | * | ** | * | * | * | ******** |
| Ivey 2013 |  | * | * | * | ** | * | * | * | ******** |
| Jacques 2013 | * | * | * | * | ** | * | * | * | ********* |
| Jacques 2015 | * | * |  | * | ** | * | * | * | ******** |
| Lajous 2016 | * | * | * | * | ** | * | * | * | ********* |
| McCullough 2012 | * | * |  | * | ** | * | * | * | ******** |
| Mursu 2008 | * | * | * | * | ** | * | * | * | ********* |
| Parmenter 2021 | * | * | * | * | ** | * | * | * | ********* |
| Ponzo 2015 | * | * |  |  | ** | * |  | * | ****** |
| Tresserra-Rimbau 2014 | * | * | * |  | ** | * | * | * | ******** |
| Tresserra-Rimbau 2016 | * | * |  | * | ** | * | * | * | ******** |
| Wedick 2012 |  | * | * | * | ** |  |  |  | ***** |
| Yan 2024 | * | * | * |  | ** | * | * | * | ******** |
| Zamora-Ros 2013 | * | * |  |  | ** | * | * | * | ******* |
| Zamora-Ros 2013 | * | * | * | * | ** | * | * | * | ********* |
| Zhao 2023 | * | * | * | * | ** | * | * |  | ******** |

# **Supplemental Table 4.** Results from prospective cohort studies examining anthocyanin

| **Outcomes and**  **Anthocyanin types** | **N Studies (N Subjects)** | **Highest vs. Lowest intake**  **Summery Estimate**  **(95% CI)** | **Heterogeneity** |
| --- | --- | --- | --- |
|  |  |  |  |
| **CHD+MI** |  |  |  |
| Total Anthocyanin | 5 studies (526,865) | **RR= 0.82 (0.70, 0.94)** | **I^2^ = 53.6%** |
| **CVD Incidence** |  |  |  |
| Total Anthocyanin | 4 studies (92,126) | **RR = 0.74 (0.54, 0.93)** | **I^2^ = 80.6%** |
| **CVD mortality** |  |  |  |
| Total Anthocyanin | 10 studies (561,764) | **RR = 0.91 (0.89, 0.94)** | **I^2^ = 14.7%** |
| **Hypertension** |  |  |  |
| Total Anthocyanin | 6 studies (182,097) | **RR = 0.92 (0.88, 0.96)** | **I^2^ = 33.1%** |
| **Stroke** |  |  |  |
| Total Anthocyanin | 6 studies (271,457) | RR = 0.88 (0.74, 1.03) | I^2^ = 80.0% |
| **Ischemic stroke** |  |  |  |
| Total Anthocyanin | 5 studies (187,299) | RR = 0.93 (0.87, 1.00) | I^2^ = 0.0% |
| **Type 2 Diabetes** |  |  |  |
| Total Anthocyanin | 8 studies (235,064) | **RR = 0.89 (0.82, 0.95)** | **I^2^ = 46.9%** |

N = number; NA = not applicable; p-y = person years; RR = relative risk

Effects sizes that are statistically significant are listed in boldface font.

# **Supplemental Figure 1.** The effect of anthocyanins on CVD mortality in prospective cohort studies

# **Supplemental Figure 2.** The effect of anthocyanins on CVD in prospective cohort studies

# **Supplemental Figure 3.** The effect of anthocyanins on CHD+MI in prospective cohort studies

# **Supplemental Figure 4.** The effect of anthocyanins on Type 2 Diabetes in prospective cohort studies

# **Supplemental Figure 5.** The effect of anthocyanins on Hypertension in prospective cohort studies

# **Supplemental Figure 6.** Subgroup analysis of female cohorts on the effect of anthocyanins on Hypertension in prospective cohort studies

# **Supplemental Table 5.** GRADE overall quality for outcomes from prospective cohort studies

| **Grading Element** | **Number of cohorts** | **Consistency** | **Precision** | | **Quality** | **Directness** | **Generalizability** | **Overall Grade** |
| --- | --- | --- | --- | --- | --- | --- | --- | --- |
| **CVD** | 4 | Moderate | Moderate | | Strong | Not assignable | Moderate | Moderate |
| **CVD Mortality** | 8 | Limited | Limited | | Strong | Not assignable | Moderate | Limited |
| **CHD/MI** | 5 | Strong | Moderate | | Strong | Not assignable | Moderate | Moderate |
| **Type 2 Diabetes** | 8 | Limited | Limited | Strong | | Not assignable | Strong | Limited |
| **Hypertension** | 6 | Moderate | Limited | Strong | | Not assignable | Moderate | Limited |

# **Supplemental Table 6:** Risk of bias assessment of Randomized Controlled Trials

| **Author Year** | **Domain 1: Risk of bias arising from randomization process** | **Domain S (crossover studies- specific): Risk of bias arising from period and carryover effects** | **Domain 2: Risk of bias due to deviations from the intended interventions (effect of assignment to intervention)** | **Domain 3: Risk of bias due to missing outcome data** | **Domain 4: Risk of bias in measurement of the outcome** | **Domain 5: Risk of bias in selection of the reported result** | **Overall risk of bias** |
| --- | --- | --- | --- | --- | --- | --- | --- |
| **Ahles 2024** | Low | Low | Some concern | Low | Low | Low | Some concern |
| **Alqurashi 2016** | Low | Some concern | Some concern | Low | Low | Some concern | Some concern |
| **Arbizu 2023** | Some concern | NA | Some concern | Low | Low | Low | Some concern |
| **Arisi 2022** | Low | NA | Low | Low | Low | Low | Low |
| **Bakuradze 2019** | Low | Low | Low | Low | Low | Low | Low |
| **Barnes 2020** | Some concern | Low | Low | Low | Low | Low | Some concern |
| **Castro-Acosta 2016** | Low | Low | Low | Low | Low | Low | Low |
| **Cavezzi 2022** | Low | NA | Some concern | Low | Low | Low | Some concern |
| **Cheatham 2016** | Low | NA | Low | Low | Low | Low | Low |
| **Chiu 2022** | Low | Low | Low | High | Low | Low | High |
| **Chung 2023** | Low | NA | Low | Low | Low | Low | Low |
| **Curtis 2009** | Low | NA | High | Low | Low | Low | High |
| **DelBó 2013** | Some concern | Low | Low | Low | Low | Low | Some concern |
| **Dohadwala 2010** | Low | Low | Low | Low | Low | Low | Low |
| **doRosario 2021** | Low | Low | Low | Low | Low | Low | Low |
| **Franck 2020** | High | NA | High | Low | Low | Low | High |
| **Guo 2020** | Low | NA | Some concern | Low | Low | Low | Some concern |
| **Heiss 2022** | Some concern | NA | Some concern | Low | Low | Low | Some concern |
| **Hillman 2022** | Some concern | NA | High | Low | Low | Low | High |
| **Hollands 2018** | Some concern | Low | High | Low | Low | Low | High |
| **Hollis 2009** | Some concern | NA | High | Low | Low | Low | High |
| **Istas 2018** | Low | Low | Low | Low | Low | Low | Low |
| **Istek 2017** | High | NA | High | Low | Low | Low | High |
| **Jeong 2016** | Low | NA | Low | Low | Low | Low | Low |
| **Johnson 2017** | Low | NA | High | Low | Low | Low | High |
| **Karlsen 2007** | Some concern | NA | Some concern | Low | Low | Low | Some concern |
| **Keane 2016** | Some concern | Low | High | Low | Low | Low | High |
| **Khan 2014** | Low | NA | Some concern | Low | Low | Low | Some concern |
| **Kimble 2021** | Low | NA | Low | Low | Low | Low | Low |
| **Kimble 2021** | Some concern | NA | Low | Low | Low | Low | Some concern |
| **Lamport 2016** | Low | Low | Low | Low | Low | Low | Low |
| **Lear 2019** | Some concern | NA | Some concern | Low | Low | Low | Some concern |
| **Lynn 2014** | Low | NA | High | Low | Low | Low | High |
| **Marniemi 2000** | Some concern | NA | Some concern | Low | Low | Some concern | Some concern |
| **Martin 2018** | Low | Low | High | High | Low | Low | High |
| **Martin 2021** | Some concern | Low | Low | Low | Low | Low | Some concern |
| **McAnulty 2014** | Some concern | NA | Low | Low | Low | Low | Some concern |
| **Nosal 2023** | Low | NA | High | Low | Low | Some concern | High |
| **Okamoto 2020** | Some concern | Low | Low | Low | Low | Low | Some concern |
| **Quesada-Morua 2020** | Some concern | Low | Low | Low | Low | Low | Some concern |
| **Rahn 2023** | High | NA | High | Low | Low | Low | High |
| **Rebello 2015** | Low | NA | High | Low | Low | Low | High |
| **Richter 2017** | High | Low | High | Low | Low | Low | High |
| **Rodriguez-Mateos 2013** | Low | Some concern | Low | Low | Low | Low | Some concern |
| **Rodriguez-Mateos 2014** | Some concern | Some concern | Low | Low | Low | Low | Some concern |
| **Rodriguez-Mateos 2016** | Low | Low | Low | Low | Low | Low | Low |
| **Rodriguez-Mateos 2019** | Low | Low | Low | Low | Low | Low | Low |
| **Rodriguez-Mateos 2019** | Low | NA | Low | Low | Low | Low | Low |
| **Rousseau 2021** | Low | NA | High | Some concern | Low | Low | High |
| **Ruel 2013** | Some concern | Low | Low | Low | Low | Low | Some concern |
| **Santhakumar 2015** | Low | Some concern | Low | Low | Low | Low | Some concern |
| **Sari 2018** | Some concern | NA | Some concern | Low | Low | Some concern | Some concern |
| **Sinclair 2022** | Some concern | NA | Low | Low | Low | Low | Some concern |
| **Solverson 2018** | High | Low | High | Low | High | High | High |
| **Stote 2017** | High | Low | Low | Low | Low | Low | High |
| **Thompson 2017** | Low | Some concern | Low | Low | Low | Low | Some concern |
| **Thompson 2017** | Low | Some concern | Low | Low | Low | Low | Some concern |
| **Tjelle 2015** | Some concern | NA | High | Some concern | Low | Low | High |
| **Toaldo 2016** | Some concern | Low | High | Low | Low | Low | High |
| **Traustadóttir 2009** | Low | Low | Some concern | Low | Low | Low | Some concern |
| **Tucakovic 2018** | Low | Low | Low | Low | Low | Low | Low |
| **Wang 2022** | Low | Low | Low | Low | Low | Low | Low |
| **Winham 2007** | Some concern | Low | High | Low | Low | Low | High |
| **Woźniewicz 2018** | Low | NA | Some concern | Low | Low | Low | Some concern |
| **Zunino 2012** | Some concern | Some concern | Some concern | Low | Low | Some concern | Some concern |

NA: Not applicable

# **Supplemental Table 7.** Results from anthocyanin randomized controlled trials meta-analyses

| **Outcomes and subgroup analyses** | **N Studies (N Subjects)** | **Net change (95% CI)** | **Heterogeneity** | |  |
| --- | --- | --- | --- | --- | --- |
| **AIx** |  |  |  | |  |
| Postprandial | 4 studies (80) | 0.22 (-2.72, 3.16) | I^2^ = 13.6% | |  |
| Chronic | 5 studies (239) | -0.88 (-2.87, 1.11) | I^2^ = 0.0% | |  |
| Cyanidin- Postprandial | 3 studies (70) | -0.89 (-3.86, 2.07) | I^2^ = 0.0% | |  |
| Cyanidin- Chronic | 4 studies (185) | -1.31 (-3.51, 0.88) | I^2^ =0.0% | |  |
| **Apo A1** | 6 studies (274) | 0.08 (-0.24, 0.39) | I^2^ =0.0% | |  |
| **Apo B** | <3 studies | **Meta-analysis not done** | | |  |
| **BMI** | 17 studies (679) | -0.08 (-0.31, 0.16) | I^2^ = 0.0 | |  |
| ≥8 weeks | 7 studies (341) | -0.09 (-0.66, 0.47) | I^2^ = 0.0 | |  |
| ≥12 weeks | 5 studies (244) | -0.11 (-0.70, 0.48) | I^2^ = 0.0% | |  |
| Blueberries | 4 studies (172) | -0.49 (-1.88, 0.89) | I^2^ = 0.0% | |  |
| Cyanidin | 10 studies (386) | -0.06 (-0.31, 0.20) | I^2^ = 0.0% | |  |
| Cyanidin- ≥12 weeks | 4 studies (168) | -0.11 (-0.85, 0.63) | I^2^ = 0.0% | |  |
| Cyanidin- cherries | 3 studies (100) | -0.23 (-1.51, 1.06) | I^2^ = 0.0% | |  |
| Delphinidin | 9 studies (406) | -0.07 (-0.32, 0.18) | I^2^ = 0.0% | |  |
| Delphinidin- ≥12 weeks | 3 studies (139) | -0.07 (-0.71, 0.56) | I^2^ = 0.0% | |  |
| Malvidin | 4 studies (172) | -0.49 (-1.88, 0.89) | I^2^ = 0.0% | |  |
| Malvidin- ≥6 weeks | 3 studies (128) | -0.21 (-2.00, 1.58) | I^2^ = 0.0% | |  |
| **Body fat** | 8 studies (334) | -0.29 (-1.35, 0.77) | I^2^ = 0.0% | |  |
| ≥6 weeks | 5 studies (192) | -0.37 (-1.55, 0.80) | I^2^ = 0.0% | |  |
| ≥12 weeks | 3 studies (113) | -0.42 (-1.63, 0.79) | I^2^ = 0.0% | |  |
| Blueberries | 3 studies (123) | -0.11 (-3.12, 2.89) | I^2^ = 0.0% | |  |
| Cyanidin | 5 studies (211) | -0.31 (-1.44, 0.81) | I^2^ = 0.0% | |  |
| Cyanidin- ≥12 weeks | 3 studies (113) | -0.42 (-1.63, 0.79) | I^2^ = 0.0% | |  |
| Cyanidin- cherries | 3 studies (148) | 0.23 (-2.23, 2.68) | I^2^ = 0.0% | |  |
| Delphinidin | 4 studies (161) | -0.44 (-1.62, 0.74) | I^2^ = 0.0% | |  |
| Malvidin | 3 studies (123) | -0.11 (-3.12, 2.89) | I^2^ = 0.0% | |  |
| **Body weight** | 17 studies (644) | -0.17 (-1.62, 1.28) | I^2^ = 0.0% | |  |
| ≥8 weeks | 7 studies (362) | -0.07 (-2.09, 1.96) | I^2^ = 0.0% | |  |
| ≥12 weeks | 3 studies (156) | -0.88 (-4.74, 2.97) | I^2^ = 0.0% | |  |
| Anthocyanin supplement | 4 studies (135) | 0.09 (-2.34, 2.52) | I^2^ = 0.0% | |  |
| Blueberries | 3 studies (128) | -0.41 (-6.04, 5.22) | I^2^ = 0.0% | |  |
| Cyanidin | 10 studies (356) | 0.00 (-1.99, 2.00) | I^2^ = 0.0% | |  |
| Cyanidin- ≥8 weeks | 4 studies (198) | 0.08 (-2.68, 2.84) | I^2^ = 0.0% | |  |
| Delphinidin | 6 studies (286) | -0.31 (-2.84, 2.22) | I^2^ = 0.0% | |  |
| Malvidin | 4 studies (185) | 0.40 (-2.86, 3.65) | I^2^ = 0.0% | |  |
| **DBP** | 21 studies (920) | -0.68 (-1.52, 0.17) | I^2^ = 0.0% | |  |
| ≥8 weeks | 11 studies (505) | -0.40 (-1.44, 0.65) | I^2^ = 0.0% | |  |
| ≥12 weeks | 7 studies (299) | 0.06 (-1.33, 1.44) | I^2^ = 0.0% | |  |
| Cyanidin | 15 studies (663) | -0.48 (-1.50, 0.55) | I^2^ = 0.0% | |  |
| Cyanidin- ≥12 weeks | 5 studies (220) | 0.01 (-1.72, 1.75) | I^2^ = 13.3% | |  |
| Delphinidin | 9 studies (390) | -0.10 (-1.43, 1.24) | I^2^ = 18.5% | |  |
| Delphinidin- ≥12 weeks | 3 studies (117) | -0.19 (-2.99, 2.61) | I^2^ = 57.0% | |  |
| Malvidin | 5 studies (193) | -1.25 (-3.70, 1.20) | I^2^ = 3.1% | |  |
| **Endothelin-1** | <3 studies | **Meta-analysis not done** | | |  |
| **Fibrinogen** | <3 studies | **Meta-analysis not done** | | |  |
| **FMD** |  |  | |  | |
| **Postprandial** | **7** **studies (119)** | **1.40 (1.11, 1.69)** | **I^2^ = 6.0%** | |  |
| **Chronic** | **6 studies (308)** | **1.59 (1.05, 2.14)** | **I^2^ = 21.2%** | |  |
| **Malvidin- Postprandial** | **3 studies (60)** | **1.69 (1.20, 2.18)** | **I^2^ = 0.0%** | |  |
| **Delphinidin-Postprandial** | **3 studies (60)** | **1.69 (1.20, 2.18)** | **I^2^ = 0.0%** | |  |
| **Cyanidin- Chronic** | **4 studies (214)** | **1.62 (1.21, 2.04)** | **I^2^ = 0.0%** | |  |
| **Delphinidin- Chronic** | **3 studies (158)** | **1.59 (1.05, 2.14)** | **I^2^ = 21.2%** | |  |
| **Glucose** | 22 studies (927) | -0.01 (-0.08, 0.06) | I^2^ = 50.6% | |  |
| ≥8 weeks | 8 studies (448) | 0.01 (-0.10, 0.12) | I^2^ = 32.2% | |  |
| ≥12 weeks | 5 studies (287) | 0.07 (-0.08, 0.23) | I^2^ = 36.1% | |  |
| Blueberries | 6 studies (257) | 0.11 (-0.09, 0.31) | I^2^ = 76.5% | |  |
| Cyanidin | 12 studies (490) | -0.04 (-0.14, 0.06) | I^2^ = 34.5% | |  |
| Cyanidin- ≥12 weeks | 3 studies (157) | 0.03 (-0.18, 0.25) | I^2^ = 58.5% | |  |
| Delphinidin | 11 studies (534) | 0.00 (-0.12, 0.11) | I^2^ = 65.4% | |  |
| Delphinidin- ≥8 weeks | 4 studies (243) | -0.03 (-0.18, 0.12) | I^2^ = 27.7% | |  |
| Malvidin | 6 studies (257) | 0.11 (-0.09, 0.31) | I^2^ = 76.5% | |  |
| **HbA1c** | 6 studies (290) | 0.00 (0.00, 0.00) | I^2^ = 0.0% | |  |
| ≥8 weeks | 3 studies (152) | 0.00 (0.00, 0.00) | I^2^ = 0.0% | |  |
| Cyanidin | 4 studies (187) | 0.02 (-0.08, 0.13) | I^2^ = 0.0% | |  |
| **HOMA-IR** | 7 studies (312) | -0.01 (-0.09, 0.07) | I^2^ = 0.0% | |  |
| ≥8 weeks | 4 studies (211) | -0.12 (-0.32, 0.08) | I^2^ = 0.0% | |  |
| Blueberries | 3 studies (122) | 0.01 (-0.08, 0.09) | I^2^ = 0.0% | |  |
| Cyanidin | 3 studies (126) | -0.09 (-0.30, 0.13) | I^2^ = 0.0% | |  |
| Delphinidin | 4 studies (186) | 0.00 (-0.09, 0.09) | I^2^ = 0.0% | |  |
| Malvidin | 3 studies (122) | 0.01 (-0.08, 0.09) | I^2^ = 0.0% | |  |
| **HDL** | 24 studies (1146) | 0.06 (-0.03, 0.15) | I^2^ = 89.2% | |  |
| ≥8 weeks | 12 studies (604) | 0.02 (-0.02, 0.06) | I^2^ = 0.0% | |  |
| ≥12 weeks | 7 studies (350) | 0.03 (-0.03, 0.09) | I^2^ = 0.0% | |  |
| Anthocyanin supplement | 3 studies (180) | 0.24 (-0.21, 0.68) | I^2^ = 94.8% | |  |
| Blueberries | 3 studies (157) | 0.00 (-0.10, 0.10) | I^2^ = 0.0% | |  |
| Cyanidin | 15 studies (666) | 0.06 (-0.08, 0.20) | I^2^ = 92.3% | |  |
| Cyanidin- ≥12 weeks | 5 studies (220) | 0.02 (-0.04, 0.08) | I^2^ = 0.0% | |  |
| Delphinidin | 9 studies (483) | 0.09 (-0.02, 0.20) | I^2^ = 81.8% | |  |
| Delphinidin- ≥12 weeks | 4 studies (193) | 0.05 (-0.02, 0.11) | I^2^ = 0.0% | |  |
| Malvidin | 4 studies (214) | 0.00 (-0.08, 0.08) | I^2^ = 0.0% | |  |
| **hsCRP** | 10 studies (477) | -0.01 (-0.09, 0.07) | I^2^ = 19.6% | |  |
| ≥8 weeks | 4 studies (183) | -0.26 (-0.64, 0.12) | I^2^ = 30.5% | |  |
| >250 mg | 5 studies (301) | -0.26 (-0.55, 0.03) | I^2^ = 11.6% | |  |
| Cyanidin | 7 studies (247) | 0.01 (-0.01, 0.03) | I^2^ = 0.0% | |  |
| **IL-6** | 4 studies (145) | -0.09 (-0.41, 0.24) | I^2^ = 0.0% | |  |
| Cyanidin | 5 studies (261) | -0.21 (-1.01, 0.59) | I^2^ = 71.8% | |  |
| **Insulin** | **6 studies (284)** | **-2.24 (-4.22, -0.27)** | **I^2^ = 0.0%** | |  |
| ≥8 weeks | 4 studies (211) | -2.29 (-8.45, 3.87) | I^2^ = 0.0% | |  |
| **Blueberries** | **3 studies (122)** | **-2.21 (-4.29, -0.13)** | **I^2^ = 0.0%** | |  |
| **Delphinidin** | **4 studies (186)** | **-2.32 (-4.39, -0.26)** | **I^2^ = 0.0%** | |  |
| **Malvidin** | **3 studies (122)** | **-2.21 (-4.29, -0.13)** | **I^2^ = 0.0%** | |  |
| **Isoprostanes** | <3 studies | **Meta-analysis not done** | | |  |
| **LDL** | 22 studies (892) | -0.04 (-0.11, 0.02) | I^2^ = 0.0% | |  |
| ≥8 weeks | 12 studies (604) | -0.06 (-0.13, 0.02) | I^2^ = 0.0% | |  |
| ≥12 weeks | 7 studies (350) | -0.05 (-0.14, 0.03) | I^2^ = 0.0% | |  |
| Blueberries | 3 studies (157) | -0.09 (-0.38, 0.19) | I^2^ = 0.0% | |  |
| Cyanidin | 14 studies (620) | -0.04 (-0.12, 0.03) | I^2^ = 0.0% | |  |
| Cyanidin- ≥12 weeks | 5 studies (220) | -0.06 (-0.15, 0.02) | I^2^ = 0.0% | |  |
| Delphinidin | 9 studies (483) | -0.08 (-0.16, 0.00) | I^2^ = 0.0% | |  |
| Delphinidin- ≥12 weeks | 4 studies (193) | -0.08 (-0.17, 0.01) | I^2^ = 0.0% | |  |
| Malvidin | 4 studies (214) | -0.13 (-0.36, 0.10) | I^2^ = 0.0% | |  |
| **oxLDL** | 4 studies (239) | -3.38 (-8.90, 2.13) | I^2^ = 78.6% | |  |
| ≥8 weeks | 3 studies (121) | -2.13 (-11.39, 7.13) | I^2^ = 81.9% | |  |
| **PWV**  Postprandial  Chronic | 4 studies (80)  9 studies (386) | -0.17 (-0.40, 0.05)  -0.07 (-0.28, 0.13) | I^2^ = 0.0%  I^2^ = 2.8% | |  |
| ≥200 mg for 1 week | 5 studies (188) | -0.27 (-0.54, -0.00) | I^2^ = 0.0% | |  |
| Cyanidin- Chronic | 6 studies (231) | 0.07 (-0.15, 0.29) | I^2^ = 0.0% | |  |
| **Delphinidin- Chronic** | **4 studies (169)** | **-0.33 (-0.62, -0.05)** | **I^2^ = 0.0%** | |  |
| **TNF-alpha** | 6 studies (274) | -1.23 (-2.68, 0.21) | I^2^ = 79.1% | |  |
| ≥8 weeks | 3 studies (137) | -3.78 (-9.45, 1.90) | I^2^ = 89.5% | |  |
| Cyanidin | 5 studies (253) | -1.94 (-6.25, 2.37) | I^2^ = 84.9% | |  |
| **SBP** | 22 studies (1050) | -0.13 (-1.33, 1.07) | I^2^ = 14.2% | |  |
| ≥8 weeks | 12 studies (635) | 0.17 (-1.22, 1.55) | I^2^ = 10.9% | |  |
| ≥12 weeks | 8 studies (429) | 0.23 (-2.15, 2.60) | I^2^ = 31.3% | |  |
| Cyanidin | 16 studies (793) | 0.15 (-1.36, 1.65) | I^2^ = 20.9% | |  |
| Cyanidin- ≥12 weeks | 6 studies (350) | 0.05 (-3.02, 3.13) | I^2^ = 48.4% | |  |
| Cyanidin- cherries | 4 studies (194) | -3.46 (-7.34, 0.43) | I^2^ = 0.0% | |  |
| Delphinidin | 10 studies (520) | -0.09 (-1.95, 1.77) | I^2^ = 30.6% | |  |
| Delphinidin- ≥12 weeks | 4 studies (247) | 0.19 (-3.23, 3.62) | I^2^ = 51.6% | |  |
| Malvidin | 5 studies (193) | -1.79 (-4.57, 0.99) | I^2^ = 0.0% | |  |
| **Total cholesterol** | 22 studies (1086) | -0.01 (-0.10, 0.07) | I^2^ = 0.0% | |  |
| ≥8 weeks | 10 studies (526) | -0.04 (-0.16, 0.08) | I^2^ = 0.0% | |  |
| ≥12 weeks | 5 studies (272) | 0.02 (-0.17, 0.20) | I^2^ = 0.0% | |  |
| Cyanidin | 15 studies (684) | -0.05 (-0.15, 0.05) | I^2^ = 0.0% | |  |
| Cyanidin- ≥12 weeks | 5 studies (220) | -0.06 (-0.21, 0.09) | I^2^ = 0.0% | |  |
| Delphinidin | 9 studies (493) | -0.09 (-0.21, 0.02) | I^2^ = 0.0% | |  |
| Delphinidin- ≥8 weeks | 5 studies (252) | -0.10 (-0.23, 0.03) | I^2^ = 0.0% | |  |
| Malvidin | 3 studies (160) | -0.13 (-0.39, 0.14) | I^2^ = 0.0% | |  |
| **Triglycerides** | 18 studies (852) | -0.03 (-0.09, 0.02) | I^2^ = 0.0% | |  |
| ≥8 weeks | 9 studies (487) | -0.02 (-0.11, 0.07) | I^2^ = 0.0% | |  |
| ≥12 weeks | 4 studies (233) | 0.06 (-0.15, 0.27) | I^2^ = 43.3% | |  |
| Cyanidin | 12 studies (568) | -0.05 (-0.13, 0.03) | I^2^ = 33.7% | |  |
| Cyanidin- ≥250 mg | 3 studies (143) | 0.07 (-0.22, 0.37) | I^2^ = 66.8% | |  |
| Cyanidin- ≥12 weeks | 4 studies (181) | 0.00 (-0.21, 0.21) | I^2^ = 72.3% | |  |
| Delphinidin | 7 studies (390) | -0.10 (-0.17, -0.04) | I^2^ = 0.0% | |  |
| **Delphinidin- ≥8 weeks** | **4 studies (213)** | **-0.10 (-0.17, -0.03)** | **I^2^ = 0.0%** | |  |
| Malvidin | 3 studies (160) | -0.05 (-0.22, 0.13) | I^2^ = 0.0% | |  |
| **Waist circumference** | 9 studies (381) | 0.14 (-1.44, 1.73) | I^2^ = 0.0% | |  |
| ≥8 weeks | 5 studies (252) | 0.57 (-1.55, 2.70) | I^2^ = 0.0% | |  |
| ≥12 weeks | 3 studies (155) | 0.70 (-1.63, 3.04) | I^2^ = 0.0% | |  |
| Cyanidin | 4 studies (167) | 0.95 (-1.50, 3.39) | I^2^ = 0.0% | |  |
| Delphinidin | 4 studies (193) | 0.51 (-1.75, 2.77) | I^2^ = 0.0% | |  |
| **LDL:HDL** | <3 studies | **Meta-analysis not done** | | |  |
| **TC:HDL** | <3 studies | **Meta-analysis not done** | | |  |

N = number; NA = not applicable; p-y = person years; RR = relative risk

Effects sizes that are statistically significant are listed in boldface font.

# **Supplemental Figure 7.** The effect of anthocyanins on acute FMD in randomized controlled trials

# **Supplemental Figure 8.** The effect of Malvidin on acute FMD in randomized controlled trials

# **Supplemental Figure 9.** The effect of Delphinidin on acute FMD in randomized controlled trials

# **Supplemental Figure 10.** The effect of anthocyanin on chronic FMD in randomized controlled trials

# **Supplemental Figure 11.** The effect of Delphinidin on chronic FMD in randomized controlled trials

# **Supplemental Figure 12.** The effect of anthocyanin on chronic Insulin in randomized controlled trials

# **Supplemental Figure 13.** The effect of Delphinidin on insulin in randomized controlled trials

# **Supplemental Figure 14.** Subgroup analysis of the effect of anthocyanin in blueberries/malvidin on Insulin in randomized controlled trials.

# **Supplemental Figure 15.** The effect of Delphinidin on chronic PWV in randomized controlled trials

# **Supplemental Figure 16.** The effect of Delphinidin on triglycerides in randomized controlled trials

# **Supplemental Table 8.** GRADE overall quality for outcomes from randomized controlled trials

| **Grading Element** | **Number of**  **studies** | **Consistency** | **Precision** | **Quality** | **Directness** | **Generalizability** | **Overall Grade** |
| --- | --- | --- | --- | --- | --- | --- | --- |
| **Significant Outcomes for anthocyanidin overall** | | | | | | | |
| **FMD (acute)** | 5 | Strong | Strong | Strong | Moderate | Moderate | ***Strong*** |
| **FMD (chronic)** | 5 | Strong | Moderate | Moderate | Moderate | Moderate | ***Moderate*** |
| **Insulin** | 6 | Limited | Limited | Limited | Moderate | Moderate | ***Limited*** |
| **Insulin (malvidin/blueberries)** | 3 | Limited | Limited | Limited | Strong | Moderate | ***Limited*** |
